# Combined Theoretical and Experimental Study to Unravel the Differences in Promiscuous Amidase Activity of Two Nonhomologous Enzymes

**DOI:** 10.1021/acscatal.1c02150

**Published:** 2021-06-30

**Authors:** Miquel À. Galmés, Alexander R. Nödling, Louis Luk, Katarzyna Świderek, Vicent Moliner

**Affiliations:** † Institute of Advanced Materials (INAM), Universitat Jaume I, 12071 Castellón, Spain; ‡ Cardiff Catalysis Institute, School of Chemistry, Cardiff University, Main Building, Park Place, Cardiff CF10 3AT, United Kingdom

**Keywords:** enzyme promiscuity, QM/MM, free energy surfaces, convolutional neural network, CALB, Bs2, amidase activity

## Abstract

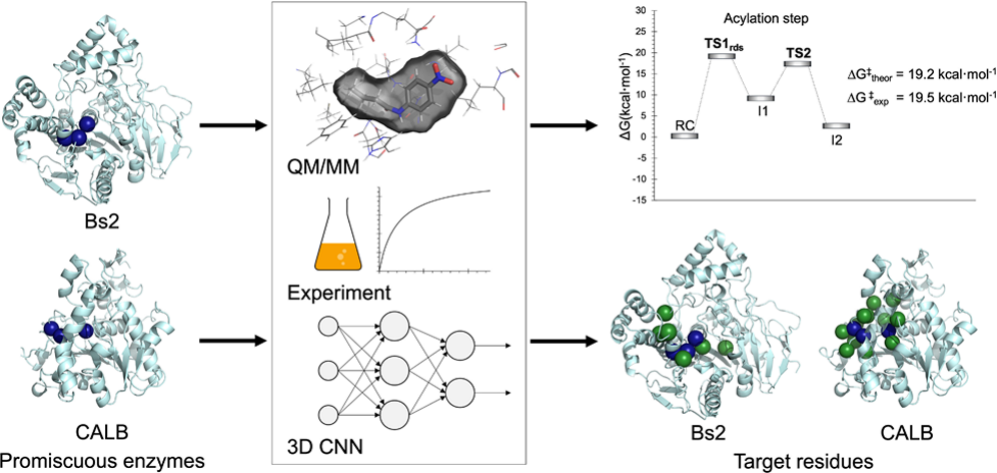

Convergent evolution has resulted in nonhomologous enzymes that contain similar active sites that catalyze the same primary and secondary reactions. Comparing how these enzymes achieve their reaction promiscuity can yield valuable insights to develop functions from the optimization of latent activities. In this work, we have focused on the promiscuous amidase activity in the esterase from *Bacillus subtilis* (Bs2) and compared with the same activity in the promiscuous lipase B from *Candida antarctica* (CALB). The study, combining multiscale quantum mechanics/molecular mechanics (QM/MM) simulations, deep machine learning approaches, and experimental characterization of Bs2 kinetics, confirms the amidase activity of Bs2 and CALB. The computational results indicate that both enzymes offer a slightly different reaction environment reflected by electrostatic effects within the active site, thus resulting in a different reaction mechanism during the acylation step. A convolutional neural network (CNN) has been used to understand the conserved amino acids among the evolved protein family and suggest that Bs2 provides a more robust protein scaffold to perform future mutagenesis studies. Results derived from this work will help reveal the origin of enzyme promiscuity, which will find applications in enzyme (re)design, particularly in creating a highly active amidase.

## Introduction

Enzyme promiscuity serves as a reservoir for new catalytic activities, playing a vital role in survival and adaptation during the course of evolution.^1,2^ Recent work by Voordeckers et al. has illustrated ancestral enzymes displayed a much wider range of substrate promiscuity at lower activity,^3^ whereas many enzymes identified to date catalyze secondary reactions whose activities are often less efficient but can be improved upon (laboratory) selection.^4^ Hence, it has been proposed that, under selective pressure (e.g., presence of new valuable chemical resources), reaction promiscuity enables the creation of new enzymes with a modified reaction profile and/or substrate specificity.^5^

From a molecular perspective, enzyme evolution takes place by modifying the electrostatic properties and geometrical complementarities of the active site such that the chemical aspects of the new molecules can be accommodated.^6−8^ This optimization process occurs gradually and smoothly in the sequence space,^9^ and closely related enzymes can act on different substrates as a consequence of divergent evolution.^10^ On the other hand, many enzymes have evolved to catalyze the same reaction whilst having no sequence homology.^11^ Such a phenomenon can be partially explained by the fact that they share similar or same active sites. Indeed, the first evidence of this convergent evolution event has been reported in the study of the serine protease family (amidase).^11−15^ Furthermore, the recruitment of ancestral enzymes that change specificity might lead to mechanistic analogues.^16^ Hence, many serine hydrolases (SHs) also contain the conserved catalytic Ser–His–Aps/Glu triad.^17^ Nevertheless, finding the proper characteristics that guide a protein or a family of proteins toward a specific activity or their capabilities to catalyze other than a primary reaction can be a challenging task. In this sense, convolutional neural networks (CNN) have been proved to be very useful in classification tasks in a wide range of fields, such as image, speech, and movement recognition, together with text analysis.^18^ Although there are expanding applications of CNN, little research has been conducted in the analysis of the protein structure.^19−22^

**Figure 1:**
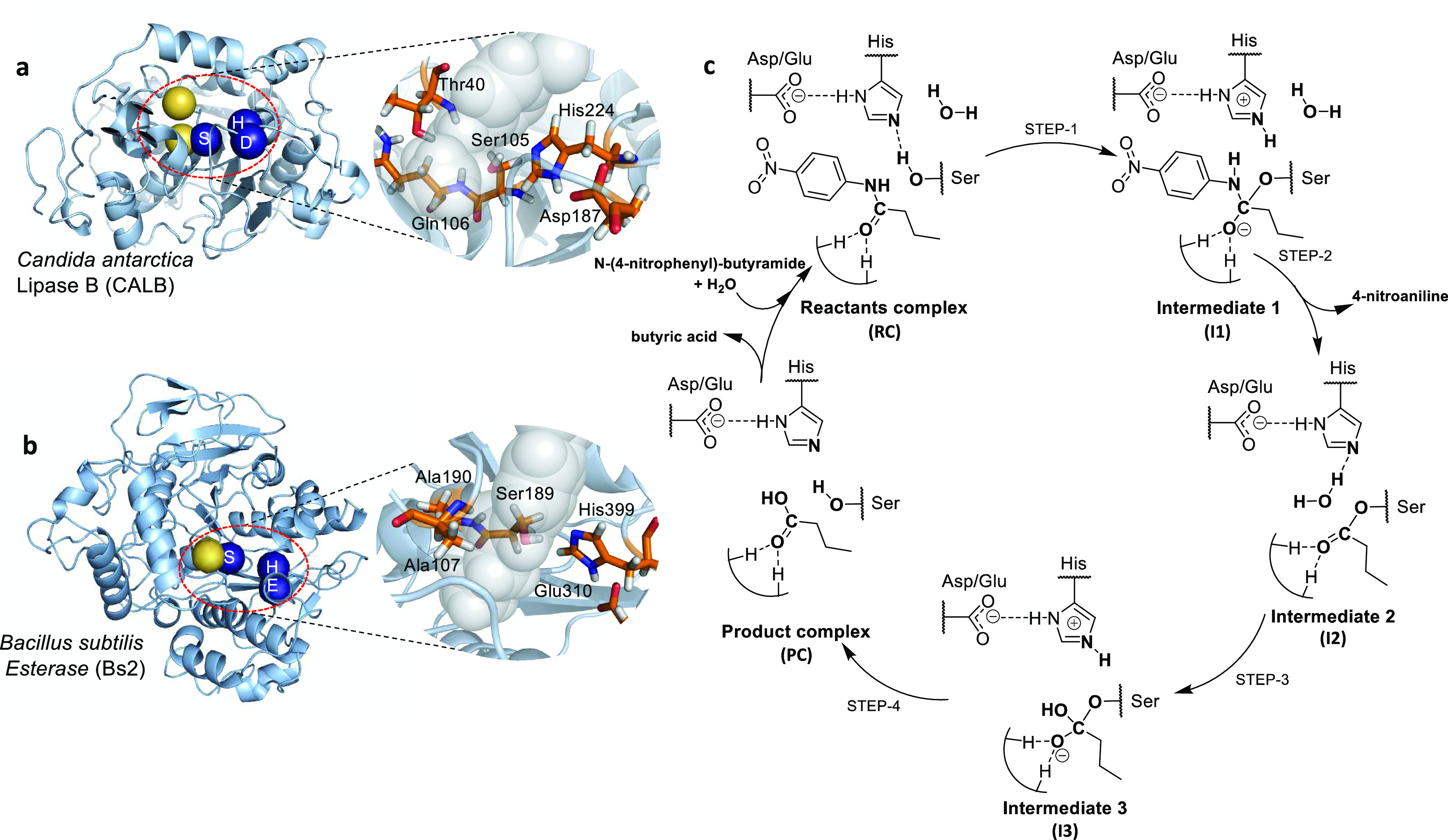
Structure and mechanism of CALB and Bs2. (a) Structure of CALB (Protein Data Bank (PDB) ID 1TCA) with details of its active site. (b) Structure of Bs2 (PDB ID 1QE3) with details of its active site. Blue spheres in panels (a) and (b) represent the catalytic triad, while yellow spheres indicate the oxyanion hole residues. (c) Schematic representation of the hydrolysis of *N*-(4-nitrophenyl)-butyramide catalyzed by CALB/Bs2. The reaction yields a molecule of 4-nitroaniline (second step, intermediate 2 (I2)) and butyric acid (last step, product complex).

*para*-Nitrobenzyl (PNB) esterase from *Bacillus subtilis* (Bs2) and lipase B from *Candida antarctica* (CALB) are nonhomologous enzymes belonging to the functional family EC 3.1.1, which accounts for carboxylic ester hydrolases. Bs2 can be further subclassified as a carboxylesterase (EC 3.1.1.1) according to the Brenda database,^23^ whereas CALB belongs to the lipase family (EC 3.1.1.3). Bs2 and CALB share only 11% of identity (see the Supporting Information) and originate in organisms that belong to a different taxonomical rank, with the former from a prokaryote whereas the latter from a eukaryote. Despite the distant relation, they have been found to catalyze similar promiscuous reactions including hydrolysis of *para*-nitrophenyl-butyrate^24−26^ and poly(ethylene terephthalate).^27,28^ Furthermore, both enzymes contain a characteristic triad whereby Ser serves as a nucleophile, His as an acid/base residue, and Asp (in Bs2)/Glu (in CALB) as a base to modulate the p*K*_a_ of the histidine residue (Figure 1). Moreover, the presence of an oxyanion hole in proximity to the catalytic triad permits the proper placement of the carbonyl oxygen of the tetrahedral intermediate in the active site. Interestingly, both enzymes have a promiscuous reaction behavior; we have recently confirmed that CALB can catalyze the hydrolysis of *N*-(4-nitrophenyl)-butyramide,^26^ while previous works showed that Bs2 is capable to catalyze identical processes.^24,25^ This has encouraged us to study the amidase activity of Bs2 in an aim to find similarities in these nonhomologous enzymes.

We envisioned that the concept of convergent evolution of enzyme promiscuity can be applied in enzyme (re)design. This must take into account that mutations in both the active site and distal location are crucial to create an active enzyme variant.^29^ To put this concept forward into an application, mechanistic studies are essential. In particular, it remains unclear if there is any parallelism in the preorganization of the active site that drives their catalytic activity in an analogous manner. Hence, hydrolysis of *N*-(4-nitrophenyl)-butyramide catalyzed by Bs2 (see Figure 1) was analyzed, employing theoretical methods based on multiscale quantum mechanics/molecular mechanics (QM/MM) and kinetic measurements. The results were analyzed and compared with those derived from our previous study on CALB,^26^ including the energetics, geometries, and electrostatic properties obtained from the full QM/MM free energy landscape of the most favored reaction paths. Deep CNN approach was performed complementarily to analyze protein residues that are conserved during the evolution of the two convergently related enzymes, generating a map of structural determinants in the vicinity of the active site.

## Methods

### Computational Methods

Wild-type *p*-nitrobenzyl (PNB) esterase sequence from *B. subtilis* (with ID P37967) was initially taken from UniProt.^30^ Because the crystal structure for this specific variant is lacking, a model was prepared based on the reported structure of PNB esterase isolated from a different organism (Bs2; PDB ID: 1QE3),^31^ and the missing residues and required mutations were introduced using Modeller^32^ (see Figure S1). A *N*-(4-nitrophenyl)-butyramide substrate was placed inside the active site pocket covalently bound to the catalytic Ser189 in the form of INT1 to avoid the possible substrate dissociation to the solvent during the equilibration molecular dynamics (MD). The protonation state of titratable residues was determined at pH 7 using the empirical program PropKa v.3.0.3.^33,34^ along with detailed inspection of the surroundings of each histidine residue. Twenty counterions (Na^+^) were placed in optimal electrostatic positions (those where the potential reaches maximum negative values) around the enzyme (further than 10.5 Å from any atom of the system and 5 Å from any other counterion, using a regular grid of 0.5 Å) to obtain the electroneutrality of the system. Finally, the protein, counterions, and the substrate *N*-(4-nitrophenyl)-butyramide were placed in a 100 × 80 × 80 Å^3^ pre-equilibrated orthorhombic box of TIP3P^35^ water molecules.

After initial energy minimizations, the system was heated to 303 K with 0.1 K temperature increment and equilibrated during short NPT MD simulations, followed by nonaccelerated classical NVT MD simulations with AMBER force field,^36^ as implemented in NAMD software.^37^ The substrate was described with the same force field parameters as determined in our previous work.^26^ During MD simulations, all atoms were free to move with periodic boundary conditions and cut-offs for nonbonding interactions. Time-dependent evolution of the root mean square deviation (RMSD) together with the *B*-factor calculation confirmed that the model was equilibrated (Figure S2).

**Figure 2:**
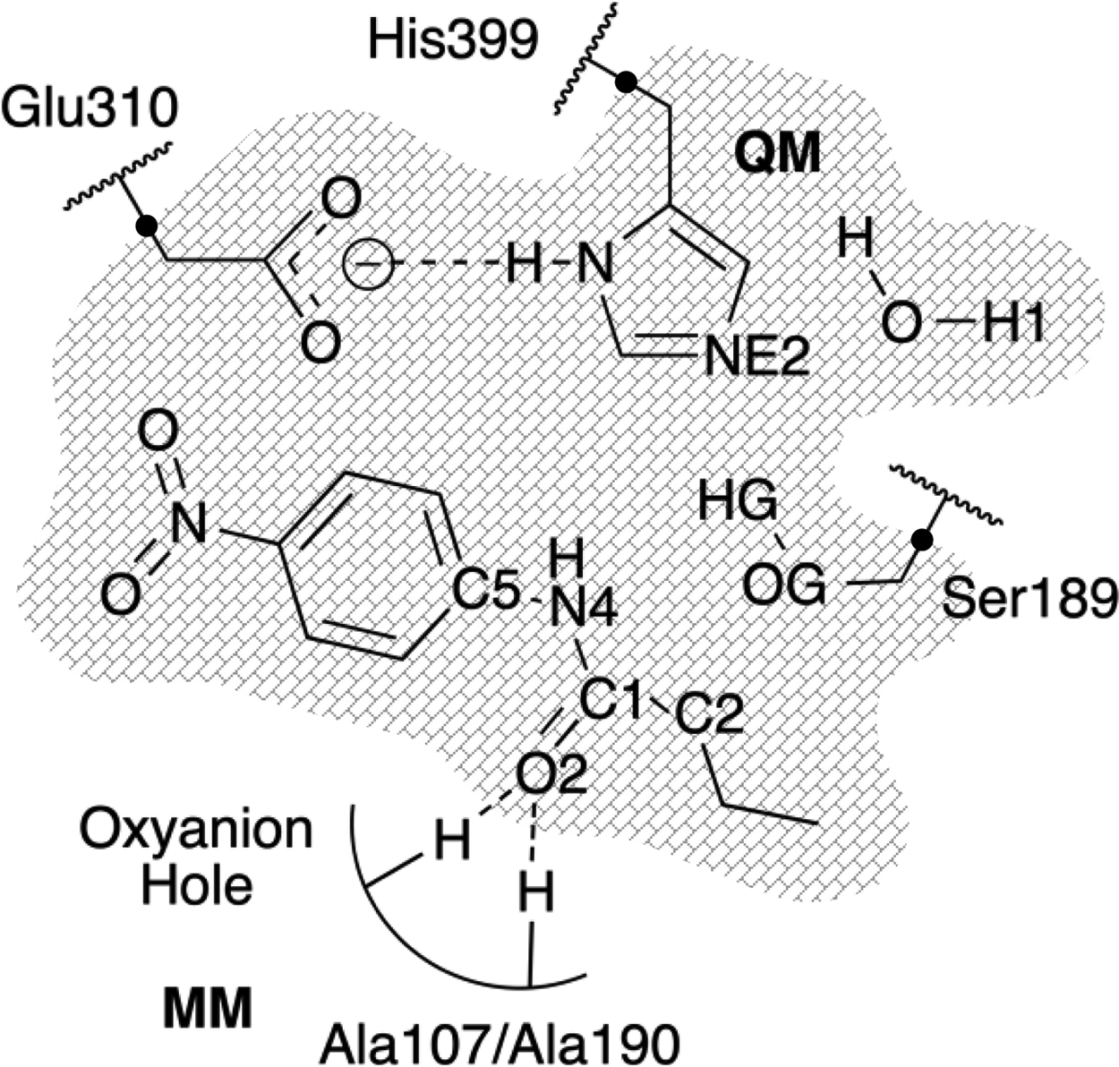
Schematic representation of the QM subset region (shadowed region). Black dots represent link atoms between QM and MM regions. The H-bond interactions between the *N*-(4-nitrophenyl)-butyramide substrate and the oxyanion hole are shown as dashed lines.

The reaction was studied using a QM/MM approach from the equilibrated structures. The most populated reactive enzyme (based on the distances of the reaction coordinates) was extracted to be used as initial geometry for subsequent calculations. The semiempirical AM1^38^ method and the M06-2X^39^ density functional were used to describe the QM subset of atoms, corresponding to the substrate and the catalytic residues of the active site (as illustrated in Figure 2). A water molecule was also included in the QM regions for the deacylation step. The optimized potentials for liquid simulations all-atom (OPLS-AA)^40^ and TIP3P^35^ classical force fields were used to treat the protein and the solvent water molecules, respectively, as implemented in fDynamo library.^41^ After potential energy surfaces (PESs) were computed, the appropriate distinguished reaction coordinates were explored, and free energy surfaces (FESs) for each of the chemical steps were calculated in terms of potentials of mean forces (PMFs) at AM1/MM level and subsequently improved at M06-2X/MM level by means of spline corrections. Structures corresponding to the stationary points were finally optimized at M06-2X/MM level and the nature of these structures as minima or saddle point of order one was verified by computing the frequency analysis. Detailed description of the computational methods can be found in the Supporting Information.

A three-dimensional (3D) CNN was built to analyze the near vicinity of the active sites in Bs2 and CALB. In particular, 11, 22, and 25 nonredundant structures from the PDB database classified as EC 3.1.1.1, EC 3.1.1.3, and EC 3.1.1.4 were extracted, respectively. A box of 20 Å was placed in the geometrical center of the catalytic residues. The box was divided into voxels of 1 Å side, so only one atom lay in a voxel. Each atom was codified using a vector with 13 features (see details in the Supporting Information), making a cube of 20 × 20 × 20 Å^3^ with 13 channels per voxel. This strategy ensured that each amino acid has a particular combination of characteristics making it unique. This also permits analogous residues, such as Asp and Glu, to be treated nonequally though they share similar chemical properties dictated by the nature of their lateral chains. Hence, to ensure that only these specific properties were seen by the network and to avoid a learning based in backbone geometry, the atoms belonging to the backbone were not taken into account. Finally, Gaussian filters were applied to the cube to resemble each atom class. One of the main limitations is the very small initial sample of structures in each family making it poorly diverse. To overcome this limitation, random rotations toward the center of the cube were applied, upsampling the dataset in this way. The initial dataset was divided into train, test, and validation datasets with a ratio of 0.7:0.15:0.15, respectively. We chose a ResNet50^42^ architecture for the training of the network, and it was build using Keras v.2.4.3^43^ and Tensorflow v.2.4.1^44^ as a backend. The alanine scan was performed mutating each residue that lies inside the box to Ala using Modeller v.9.25.^32^ Then, 1000 predictions on each mutant protein were done applying random rotations. The final classification score was measured as the difference between the ratio of correct classifications and the ratio of the most probable remaining class.

### Experimental Methods

The synthesis of *N*-(4-nitrophenyl)-butyramide was performed according to a known procedure.^26^ The production of Bs2 was based on a previously reported procedure.^24^ For the 96 well-plate kinetic assays, stock solutions of Bs2 (in 50 mM NaP_i_, pH 7.0) and *N*-(4-nitrophenyl)-butyramide (in dimethyl sulfoxide (DMSO)) were prepared. The protein stock solution was kept on ice until use and was freshly prepared before each usage. DMSO and substrate stock solution were added to wells of a 96 transparent well-plate to a total of 15 μL. Buffer (50 mM NaP_i_, pH 7.0) was added to a total volume of 135 μL (150 μL in the case of controls to monitor the substrate stability). The plate was transferred into a plate reader, double orbitally shaken for 5 s and the absorption at λ_Ex_ = 405 nm measured to check the correct substrate distribution. Then, 15 μL of protein stock solution was added to each well except the enzyme-free controls within 5 min. The final assay conditions were 150 μL volume, 10% DMSO, *N*-(4-nitrophenyl)-butyramide (10, 50, 100, 250 500, 1000, 2000, 3000 μM), and 20 μg·mL^–1^ protein. The plate was sealed with an airtight and UV–vis transparent self-adhesive plastic cover sheet. After sealing, the plate was placed into the plate reader and the assay was monitored for 14 h.

## Results and Discussion

### Bs2 vs CALB Sequence Comparison

**Figure 3:**
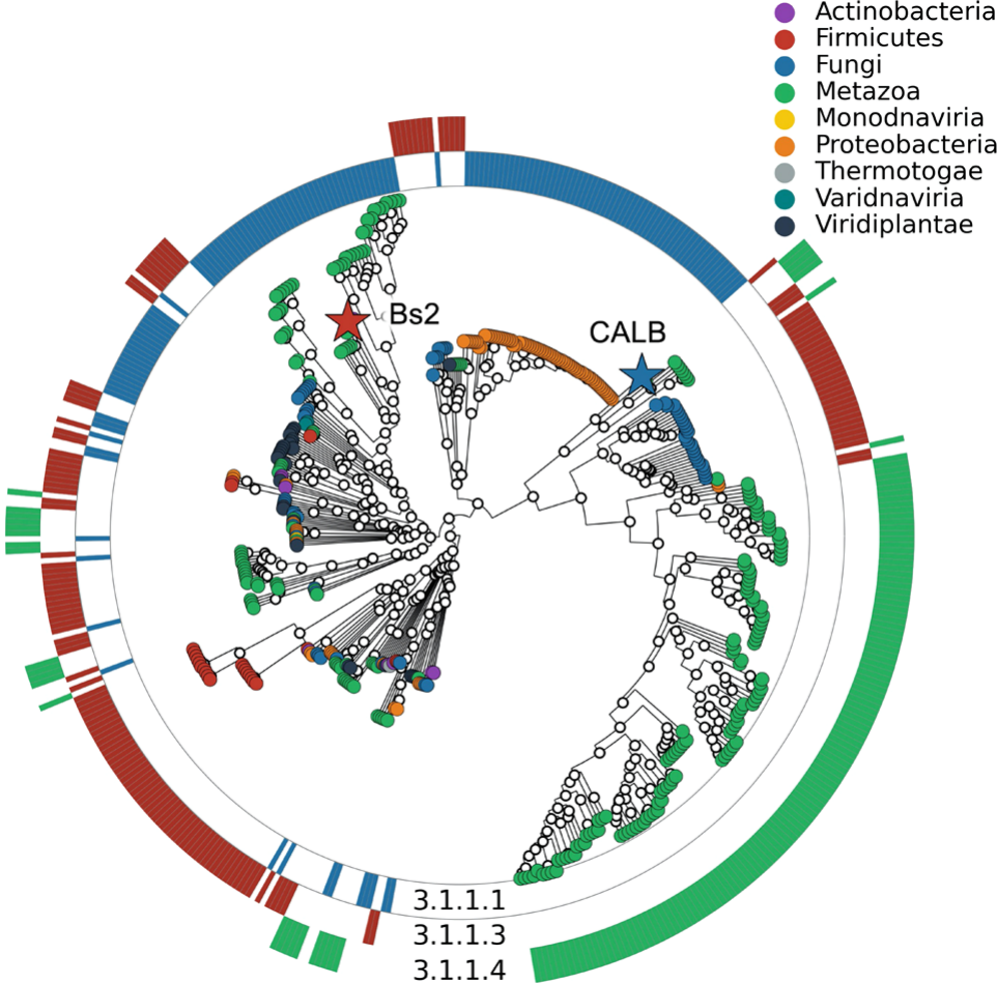
Phylogenetic tree of sample enzymes belonging to EC 3.1.1. External circles codified by each EC number studied. Enzymes were also classified by phylum. The red and blue stars correspond to Bs2 and CALB, respectively.

Bs2 and CALB are remarkably different from a structural point of view, although they present some common features such as, as commented above, a common characteristic catalytic triad formed by a serine serving as a nucleophile, a histidine that acts as an acid/base residue, and Asp/Glu as a base to modulate the p*K*_a_ of the histidine residue. Moreover, according to the CATH database,^45^ which provides a hierarchical classification of protein domains based on their folding patterns, they both belong to the α/β hydrolase superfamily. Based on the phylogenetic tree of the 450 manually selected protein sequences of EC 3.1.1.1, EC 3.1.13, and EC 3.1.1.4 deposited in Uniprot^46^ (Figure 3), it can be concluded that Bs2 and CALB are distantly related proteins converged in a function. These EC domains were taken since a reasonable number of crystallographic structures were available in the PDB database,^47^ making it possible to be used for CNN training.

### Reaction Mechanism of Bs2 as an Amidase

**Figure 4:**
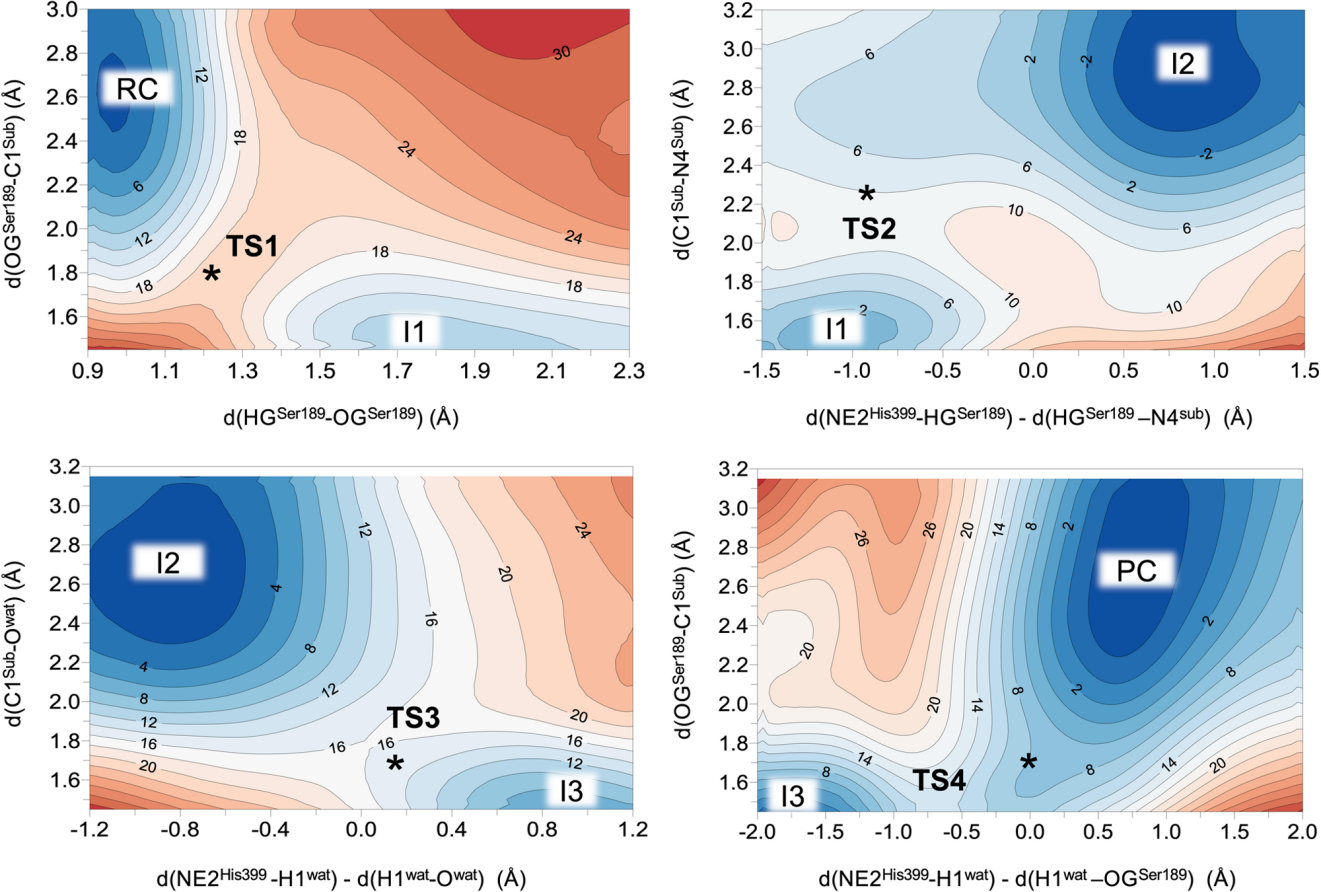
M06-2X:AM1/OPLS-AA free energy surfaces of the hydrolysis of *N*-(4-nitrophenyl)-butyramide catalyzed by wild-type Bs2. Distances are given in angstrom and isoenergetic lines are in kcal·mol^–1^. The black stars indicate the position of single transition-state (TS) structures fully optimized at M06-2X/OPLS-AA level of theory.

The hydrolytic reaction of *N*-(4-nitrophenyl)-butyramide catalyzed by Bs2 was investigated using QM/MM methodologies, assuming the mechanism previously suggested for serine hydrolases.^26,48^ Free energy surfaces (FES) of every step of the reaction were computed in terms of potentials of mean force (PMFs). The resulting FESs reveal that, as expected, the reaction takes place in four steps involving acylation of the substrate, release of the leaving group (4-nitroaniline), hydrolysis of the acyl enzyme complex, and culminating in the regeneration of the active site by final product formation (Figures 1c and 4).

**Figure 5:**
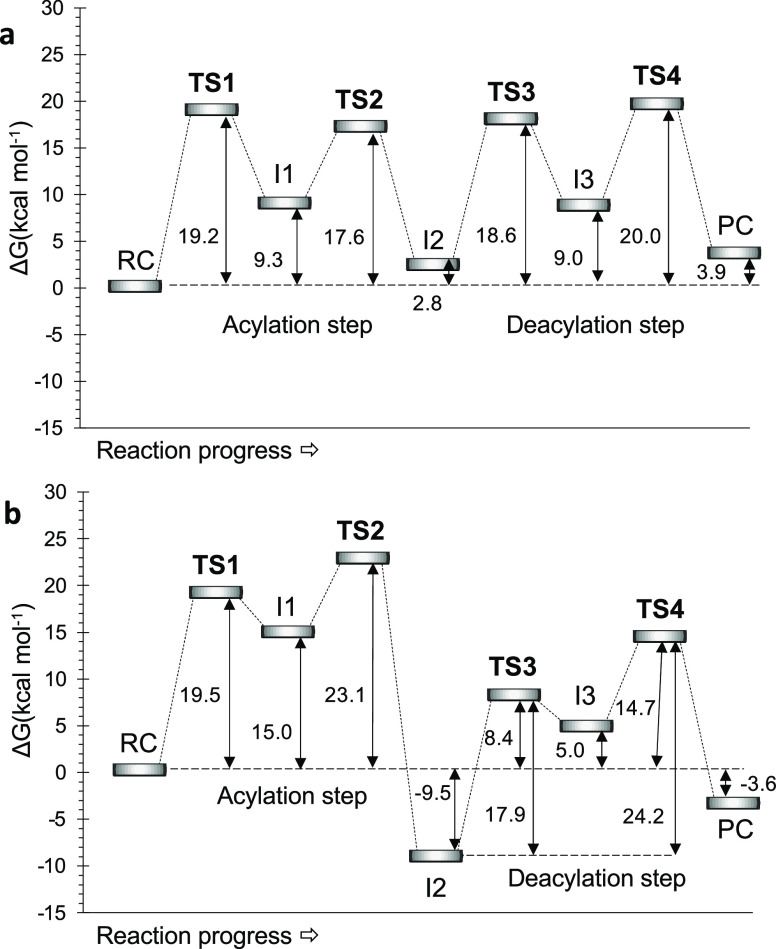
Free energy profiles of the hydrolysis of *N*-(4-nitrophenyl)-butyramide catalyzed by (a) Bs2 and (b) CALB (from ref (26)) computed at M06-2X/OPLS-AA level.

The free energy profile derived from the FESs shows that the rate-limiting step for the formation of the first product, 4-nitroaniline in I2, corresponds to the first step in which Ser198 activated by His399 attacks C1 of the substate that covalently binds to the enzyme (see Figure 5a). The computed overall energy barrier for this step was 19.2 kcal·mol^–1^. The rate-limiting step of the deacylation corresponds to the final step during which the butyric acid product is formed and the enzyme is regenerated (20.0 kcal·mol^–1^).

The predictions derived from the QM/MM computational protocol were assessed experimentally by measuring the release of 4-nitroaniline that according to the results of our calculations can sufficiently serve as a gauge for the catalytic efficiency of Bs2. As observed in Table 1, the experimentally rate constant *k*_cat_ determined by a UV–vis spectrophotometric assay was found to be 0.0185 ± 0.0007 s^–1^, which is 2 orders of magnitude higher than previously reported.^24,25^ A possible explanation for this difference could be associated with the purity of the starting material or the difference in the linker used between the enzyme and hexahistidine tag. Additionally, previous studies employed low protein concentrations (66 ng·mL^–1^)^25^ which may be prone to error during kinetic measurement. Despite these discrepancies, the rate constant obtained in our studies is in excellent agreement with the computationally assessed enzyme activity. In the frame of the transition-state theory,^49−51^ the measured kinetic rate constant corresponds to an energy barrier of 19.5 kcal·mol^–1^ at 294 K, which is very close to the computationally predicted value of 19.2 kcal·mol^–1^ obtained at 300 K.

**Table 1:** Experimental and Theoretical Kinetic Values for the Hydrolysis of *N*-(4-Nitrophenyl)-butyramide Catalyzed by Bs2 and CALB

enzyme	*k*_cat_ (10^–3^ s^–1^)	*K*_M_ (mM)	*k*_cat_/*K*_M_ (s^–1^·M^–1^)	Δ*G*^exp^ (kcal·mol^–1^) at 294 K	Δ*G*^theor^ (kcal·mol^–1^) at 300 K
Bs2	18.54 ± 0.66	0.182 ± 0.043	108 ± 27	19.5	19.2
CALB^a^	0.29 ± 0.06	4.2 ± 1.3	0.069 ± 0.026	22.7	23.1

a Results from our previous studies.^26^

Although the mechanism of the studied reaction is rather predictable and follows the classical order of chemical transformations, it is of interest to identify some features of Bs2 that allow catalysis of the same secondary reaction as in CALB. Therefore, for the rest of this work, a comparative analysis of catalytic efficiency in these two promiscuous enzymes will be presented.

### Promiscuity of Bs2 vs CALB

After analyzing the free energy profile of the reaction pathway determined for both enzymes (Figure 5), it is noticeable that the kinetics of the acylation and deacylation steps are significantly different despite the rate-limiting step of the full process corresponds to the last step in both enzymes. Comparison of the full chemical reaction shows how the overall chemical steps in Bs2 is a slightly endergonic process, while the full reaction in CALB is slightly exergonic. At this point, we must keep in mind that the reported free energy profiles correspond to total energies of the chemical system plus the full solvated proteins for the chemical steps. Both proteins can behave slightly different along the full catalytic process, until all products are released and the enzyme recovered for the next catalytic cycle. Regarding the deacylation step, the energies of TS3 and TS4 relative to the reactant complex (RC) are much higher in the Bs2 reaction in comparison to the same steps in CALB. Nevertheless, the overstabilization of intermediate 2 in CALB makes the overall free energy barrier, determined by the energy of TS4 relative to I2, much higher than in the Bs2 cycle (24.2 vs 20.0 kcal·mol^–1^, respectively). Nevertheless, keeping in mind that the kinetic experiments are based on the determination of 4-nitroaniline released in intermediate 2, a deeper analysis will be based on just the acylation process where, as mentioned above, a very good agreement was obtained between the experimental rate constants and the predicted activation free energies (see Table 1).

The computed free energy barrier of the second step, with respect to the intermediate 1 (I1), is the same in both enzymatic systems suggesting that the stabilization of this intermediate 1 dictates the final shape of the energetic profile of the acylation process (see Figure 5). A more stable I1 in Bs2 is reflected in an overall lower free energy barrier computed for the second step with respect to RC. Interestingly, the different behavior displayed by Bs2 and CALB with respect to the acylation step can be compared with recent QM/MM studies carried out in our laboratory on other proteases such as the cruzain^52^ or SARS CoV-2 M^pro^^53,54^ cysteine proteases, or the 20S subunit of proteasome,^55^ where the protonation of the amine leaving group appears to be crucial. In the present study, the difference of 5.7 kcal·mol^–1^ in the stabilization of I1 between Bs2 and CALB can be explained based on electrostatic properties (see the Supporting Information). We observed a much higher positive electrostatic potential on the carbonyl oxygen (O2) of the substrate in Bs2 than in the corresponding atom within CALB during the transition from RC to I2. This, together with the variations of the charge distribution on the substrate from RC to I1 (see a full list of computed CHelpG charges of the key atoms of the system at M06-2X/MM level in the Supporting Information), can be at the origin of the relative energies of I2: the negatively charged O2 in I1 is more stabilized in Bs2 than in CALB. Nevertheless, because a higher electrostatic potential is also measured on the NE2 atom of the catalytic histidine residue (His399 in Bs2 and His224 in CALB) from RC to I2, CALB would stabilize the positive charge that appears in the imidazole ring in I1 more effectively than Bs2 (280.3 and −132.7 kJ·mol^–1^·e^–1^ in Bs2 and CALB, respectively). Considering the final relative energies of I2 in both systems, it can be assumed that the effect on the carbonyl group of the substrate appears to be more relevant than the effect on histidine, which is in agreement with previous computational studies on the substrate promiscuity of CALB.^26^

**Figure 6:**
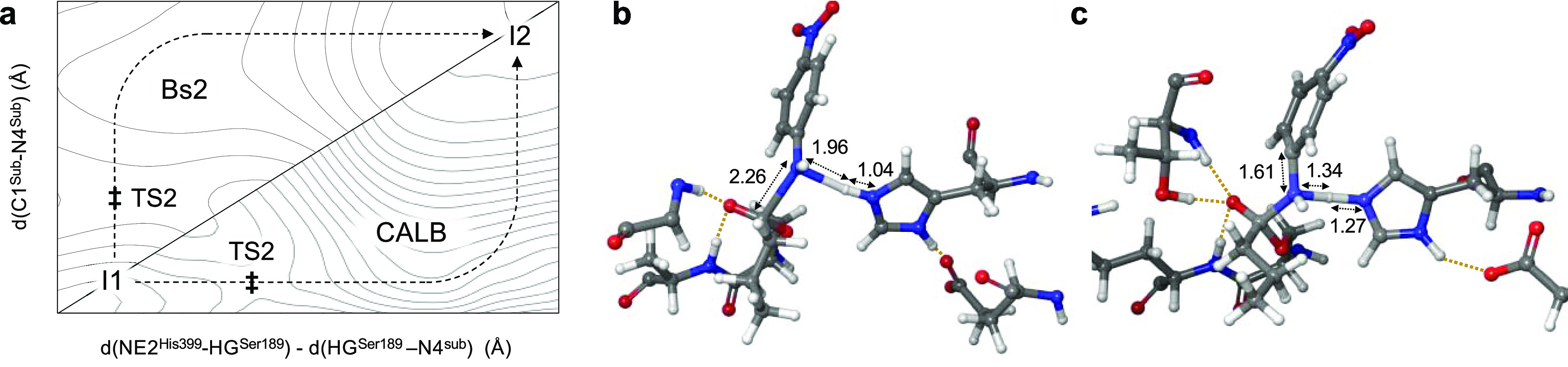
Schematic representation of the different reaction pathways of step 2 between Bs2 and CALB, with their respective transition-state structures. (a) Path followed by both enzymes on the FES of step 2. Double daggers represent the localized TS2 at M06-2X/MM level. (b) Localized TS2 at M06-2X/MM level in Bs2. (c) Localized TS2 at M06-2X/MM level in CALB.

Interestingly, subtle differences can be detected between the reaction pathways of Bs2 and CALB in the second step of the studied process. The proton transfer and C–N bond breaking occur in a concerted but asynchronous manner in both enzymes but the timing of the two events is completely different (Figure 6). In Bs2, the breaking of the C–N bond precedes the proton transfer from the catalytic His to the nitrogen of 4-nitroaniline. An inverse order of events was found in the reaction catalyzed by CALB where the transfer of the proton occurs before the breaking of the C–N bond. This geometrical analysis is in agreement with the analysis of the evolution of charges. Thus, from the CHelpG charges computed at M06-2X/MM level in TS2 (see the Supporting Information for the full list of charges), a significant difference is observed in the nitrogen N4 atom of the substrate with −0.91 and −0.49 au in Bs2 and CALB, respectively. This confirms that, while in Bs2 the breaking of the C–N clearly precedes the proton transfer and there is less negative charge built on the carbonyl oxygen O2 (−0.842 and −1.086 au in Bs2 and CALB, respectively), the more advanced proton transfer in CALB results in a more advanced double bond formation between O2 and C1 (see Figure 2 for numbering of the atoms). As suggested by the minimum energy paths traced in Figure 6, once crossing the TS2, the rest of the process involving the breaking of the C1–N4 bond is barrierless in CALB, while in Bs2 the energy barrier is mostly associated with the breaking of the amide bond.

**Figure 7:**
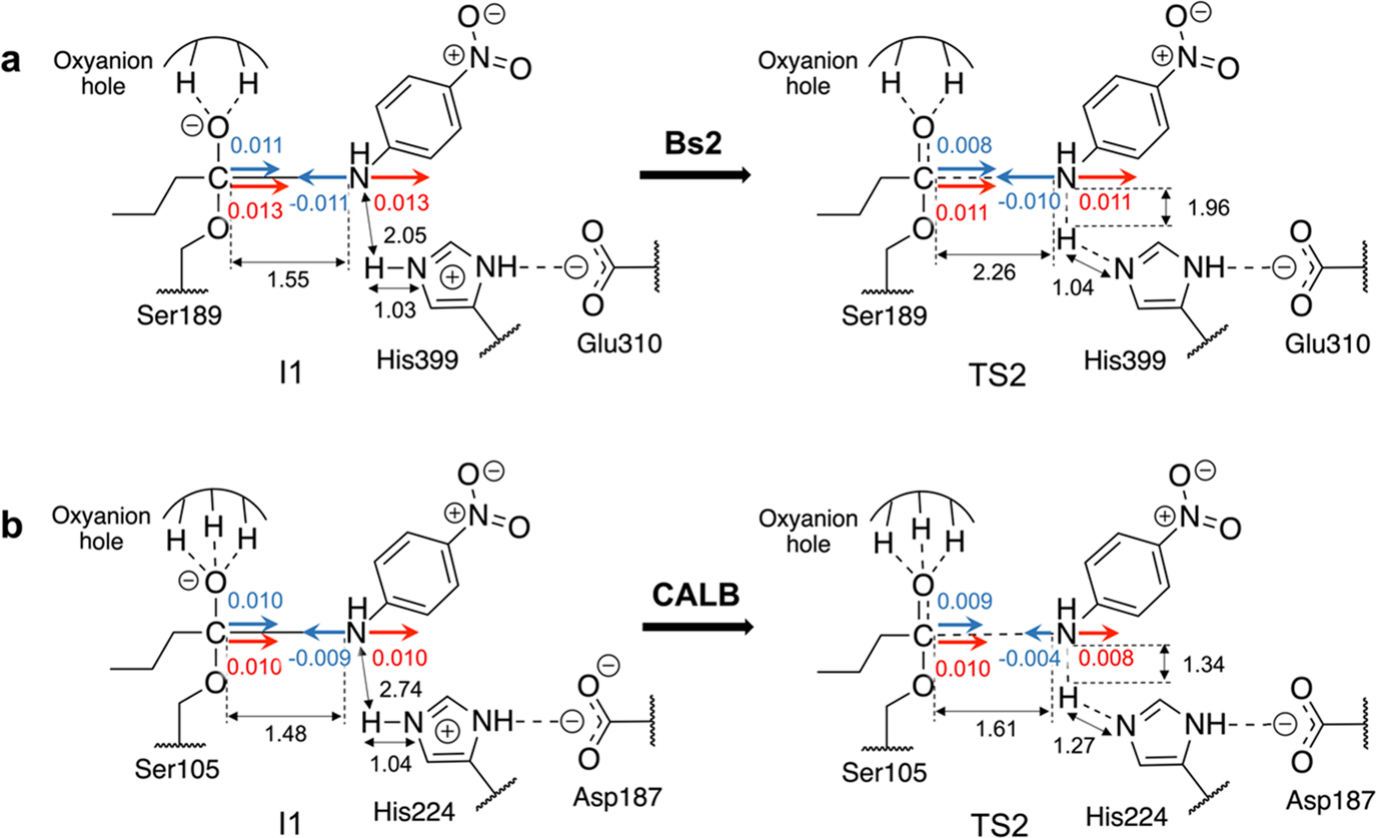
Schematic representation of the electrostatic features of the active site in (a) Bs2 and (b) CALB, in I1 and TS2 of the acylation step. Projections of the electric field created by the proteins in the C–N peptide bond direction are shown as red arrows in C1 and N4 atoms, blue arrows are the resulting electrostatic forces (given in au × 10^3^) projected on the direction of the vector defined from C1 to N4, and black arrows represent the distance between atoms in angstrom.

The difference in the mechanism of the second step can be further revealed by analysis of the electrostatic forces generated by the protein on the scissile C1–N4 bond (see Figure 7). The calculation of the electric field generated on the C and N atoms in I1 and TS2, combined with the charges computed on these atoms, shows stronger attractive forces between these two atoms in Bs2 than in CALB. Nevertheless, similar energy barriers computed from I1 are in agreement with the similar evolution of resulting electrostatic forces from I1 to TS2. Interestingly, slightly weaker attractive electrostatic forces are detected between the two atoms of the scissile bond in TS2 than in I1, in both systems.

### Three-Dimensional Convolutional Neural Network (3D CNN)

We trained a 3D CNN to test, first, whether our machine learning protocol would be able to reproduce the classification of the two enzymes into their EC number based on the surroundings of its active site. One of the most successful architectures in the Deep Learning paradigm is the ResNet^42^ for its very fast convergency and high accuracy in classification tasks. Consequently, a 3D ResNet50 to classify the proteins by EC number was used. We achieved an overall accuracy of 99.8%, implying that the network was capable to correctly classify the proteins into its functional class based on the surrounding of the active site.

**Figure 8:**
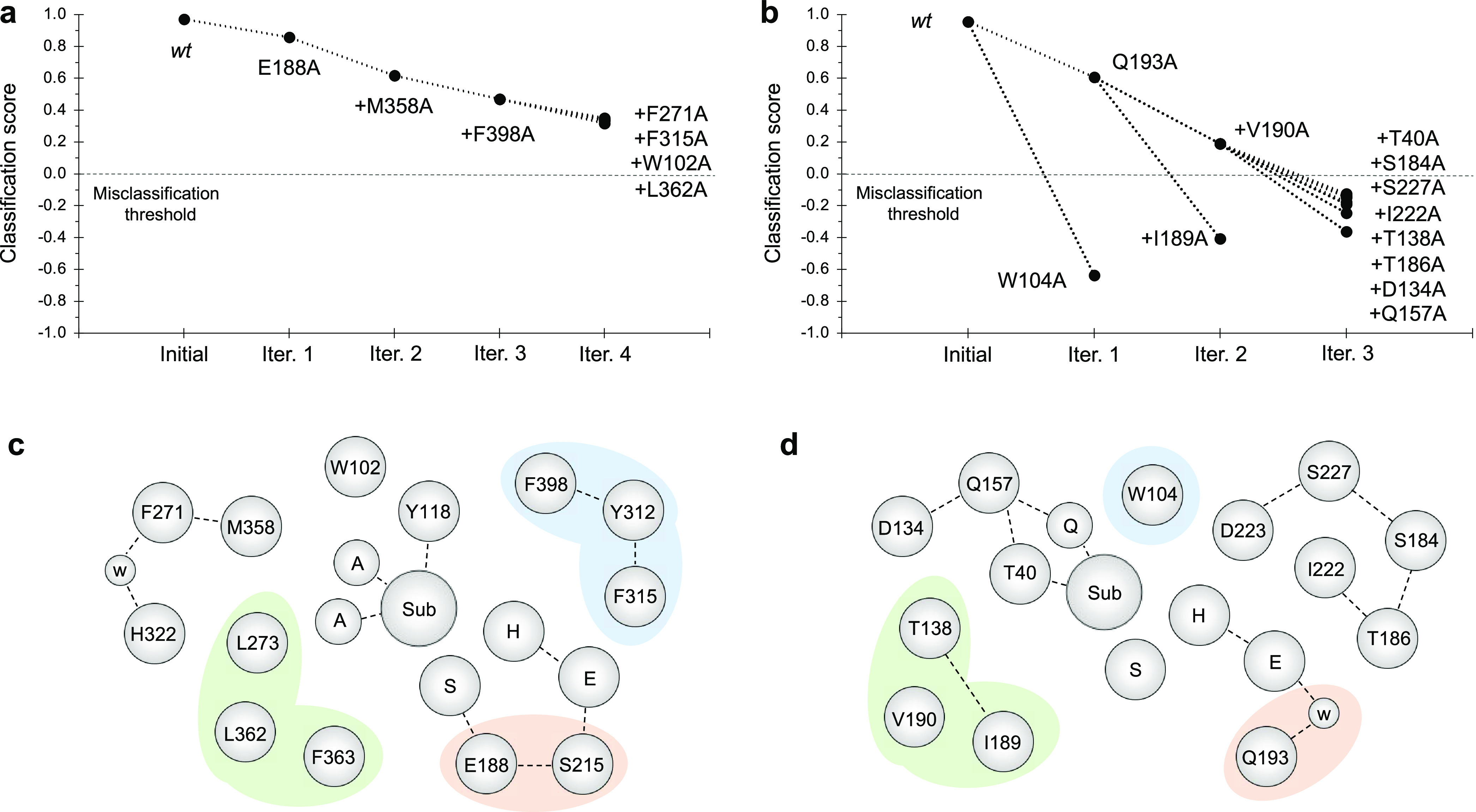
In silico CNN-mediated alanine scan in the vicinity of the active sites of Bs2 and CALB. (a) Four iterations of mutagenesis in Bs2. (b) Three iterations of mutagenesis in CALB. Previous mutations were accumulated in each iteration. (c) Schematic representation of the key residues in the vicinity of the active site in Bs2. (d) Schematic representation of the key residues in CALB. The green area corresponds to the hydrophobic acyl-acceptor pocket, blue highlights the hydrophobic cap and the ring stacking area, and the orange area corresponds to the residues involved in the stabilization of the catalytic site.

Similar to the experimental protocols, we performed an alanine scanning experiment whereby residues key to its correct classification by the CNN protocol (and hence to enzyme functionality) were identified through site-directed alanine replacement (see the Supporting Information for details). We reasoned that if the misclassification rate increases when a mutation is done to a specific residue, it might be important among the family. Performing consecutive iterations and accumulating the relevant mutations allows the identification of residues that the network has learned to be common in the family. According to the results, the network was able to classify Bs2 and CALB with its correct EC number with an accuracy of 98.1 and 99.6%, respectively. Nevertheless, it must be noted that the small initial sample of structures in each family makes this approach poorly diverse, but it can be successfully used to elucidate the common features of each family. Moreover, relevant findings from the alanine scan indicate crucial residues (see the Supporting Information for an illustration of their location) in the structure of Bs2 and CALB that define their classification in their corresponding families (Figure 8). A score of 1 corresponds to a perfectly classified protein, while a score of −1 refers to a perfectly misclassified class.

Our CNN method suggests that Glu188 is crucial to Bs2, and a detailed analysis confirms that this residue is involved in a hydrogen bonding network in the vicinity of the active site, including Ser215 and the catalytic residues Ser189 and Glu310 (see Figure 8c). Hence, Glu188 may play a role in the structural stabilization of the active site. Moreover, keeping in mind that this is a titratable residue, its protonation state, which can be modulated by the environment, may be decisive in the binding of charged substrates and the resulting electrostatics in the active site.

Other crucial networks in Bs2 involve aromatic and hydrophobic residues. The CH−π interaction between Met358 and Phe271 resulted in a water bridge interaction with a superficial His322. Accordingly, Met358 helps stabilizing the overall protein structure. Phe398 and Phe315 also form a sandwich along with Tyr312 through complex π–π interactions. These residues play a role in the stabilization of the substrate forming a π–π stacking network, as highlighted by Bornscheuer and co-workers^24^ and further confirmed by our simulations. Leu362 forms a hydrophobic pocket along with Phe363 and Leu273 where the acyl chain of the substrate is placed. This hydrophobic pocket is essential for the acceptance of acyl chain substrates. Finally, Trp102 might be involved in the stabilization of Tyr118 with a hydrogen bond established with the nitro group of the substrate.

In CALB, the mutagenesis has a considerable impact (Figure 8b). This can be explained either due to the higher diversity in the trained dataset which enables identification of more crucial features or, simply, the distinctive features of this enzyme classification. Trp104 forms a hydrophobic pocket which permits the correct placement of the substrate in the active site, similar to the role played by Phe398 and Phe315 in Bs2. Based on our findings, this residue appears to be imperative because mutation for this residue resulted in misclassification. From the localized structures at M06-2X/MM level, Ile189, Val190, and Thr138 form a hydrophobic pocket and interact with the acyl chain of the substrate (Figure 8d). Moreover, the lateral chain of Thr138 established a strong hydrogen bond with the backbone of Ile189, assisting the formation of a rigid pocket that has a similar role to the one formed by Leu362, Leu273, and Phe363 in Bs2. Our previous studies^26^ revealed that Asp134, located in proximity to the substrate, is protonated at pH 7 and it is involved in a complex hydrogen bonding network including Gln157 and the residues used in forming the oxyanion hole, Gln106, and Thr40. These interactions likely assist the formation of the oxyanion hole that is essential for the activity.

Another noteworthy residue that assists the structural stabilization of the active site is Gln193, which establishes a hydrogen bond between the catalytic Asp187 through a water bridge. This type of interaction has also been seen in Bs2 (the network of Glu188, Ser215, and Glu310). Polar residues such as Ser184 and Ser227 have been identified as their mutations resulting in misclassification of CALB after three iterations of the alanine scan. These serine residues are positioned behind the catalytic His224 and seem to be involved in the maintenance of the tertiary structure in forming hydrogen bond interactions with the backbone of residues Asp223 and Ile222. Finally, another residue identified by CNN as important is Thr40. This amino acid is part of the oxyanion hole and establishes a hydrogen bond with the carbonyl oxygen of the substrate via its lateral chain. The other two hydrogen bonds of the oxyanion hole are created by the backbone. The fact that the backbone is not represented in the CNN limits the identification of the oxyanion hole in both Bs2 and CALB. Even though limitations exist, our findings could guide further research and contribute to expanding the knowledge of the structural conformation of both enzymes.

Interestingly, some of the Bs2 residues that our CNN reveals as relevant for the structure have already been previously selected on directed evolution and mutagenesis studies. For instance, Bornscheuer and co-workers found that Glu118,^25^ Phe314, and Phe315^56^ have a very important role in the kinetic performance of Bs2. In a directed evolution study by Arnold and collaborators,^57^ despite many of the tested mutations related to the surface, the mutation of Met358 was found to improve the kinetic activity in organic solvents. Other studies have reported that Trp104 is essential and forms a hydrophobic pocket in CALB,^58−60^ while residues such as Ile189, Val190, and Thr138 have also been proven to be crucial in the kinetic performance of CALB.^60−62^

## Conclusions

The present study has focused on understanding and explaining how two structurally different enzymes, Bs2 and CALB, have evolved converged specificity to catalyze the same amidase reaction. The combined experimental and computational approach in analyzing the reaction mechanism of the hydrolysis of *N*-(4-nitrophenyl)-butyramide catalyzed by Bs2 has allowed deep comparative analysis with our previous study on the same reaction catalyzed by CALB.^26^ The first conclusion that derives from our results is the excellent agreement between our predicted free energy barriers and the experimentally determined rate constants for the acylation step of the process. Subsequently, we confirm that Bs2 and CALB catalyze the full reaction in a similar fashion, with the same rate-limiting step corresponding to the last step of the deacylation process. However, significant mechanistic differences were identified in the acylation stage. The QM/MM free energy landscape of Bs2 illustrated that the rate-limiting step for the formation of the first product, 4-nitroaniline, corresponds to the first step in which Ser198 activated by His399 attacks C1 of the substrate, forming an enzyme covalent intermediate. In contrast, in the case of CALB, the kinetics of the acylation stage are determined by the barrier of the proton transfer and the substrate C–N bond breaking occurring in the second step. Moreover, despite this second step is concerted in both enzymes, the timing of the events taking place are completely different: in Bs2, the breaking of the C–N bond precedes the proton transfer from the catalytic His to the nitrogen of the 4-nitroaniline, while the inverse order is found in the reaction catalyzed by CALB. This can be reasoned based on protein electrostatics; the charges on the C and N atoms of the scissile bond provoke a slight difference in the electrostatic forces on these two atoms. Moreover, the overall barrier difference can be explained by a more stable I1 in Bs2 due to a stabilization of the negative charge accumulated in the carbonyl oxygen atom by the electrostatic potential created by the protein. The evolutive analysis of the protein geometries using deep learning approaches such as convolutional neural networks (CNN) has proved to be useful in predicting the classification of both enzymes and confirming their significant structural differences. Three-dimensional CNN has been used to analyze the vicinity of the active sites in Bs2 and CALB, revealing residues that are decisive in their 3D structures. According to the 3D CNN results, Bs2 would be a more robust protein scaffold to perform mutagenesis studies to improve the, in this case, amidase activity without dramatically perturbing the structure of the protein. On the contrary, mutations on CALB appear to have significant effects on the 3D structure of the protein. This 3D CNN analysis can be considered a practical illustration of the use of machine learning protocols that, together with the comparative analysis of the reactivity and electrostatic effects, can be decisive to unravel the origin of the promiscuity of enzymes, which in turn can be the bedrock of new strategies in protein engineering.

## Supporting Information Available

Sequence alignments of crystal structures; details of computational and experimental methods; time dependence of RMSD of backbone atoms in the equilibration MD simulations and *B* factors; key interatomic distances and CHelpG charges of structures optimized at M06-2X/MM level; electrostatic potential and electric field created by the proteins Bs2 and CALB on key atoms in the different states along the reaction; representation of the key residues identified by the alanine scan using the CNN on both Bs2 and CALB; Cartesian coordinates of transition-state structures localized at M06-2X/MM and imaginary frequencies; map of plasmid with wild-type Bs2 gene in pET-28a vector used for recombinant protein expression; LC chromatograms; mass spectra; and Michaelis–Menten plots (PDF)
*xyz* coordinates of TS1 and TS2 of Bs2 (XYZ)
*xyz* coordinates of TS3 and TS4 of Bs2 (XYZ)

## Supplementary Material

cs1c02150_si_001.pdf

cs1c02150_si_002.xyz

cs1c02150_si_003.xyz
