# Supplementary material for: Combined Theoretical and Experimental Study to Unravel the Differences in Promiscuous Amidase Activity of Two Nonhomologous Enzymes
Source: ACS Catal. 2021 Jun 30;11(14):8635–44. doi: 10.1021/acscatal.1c02150 (PMC9299431; doi:10.1021/acscatal.1c02150)
Supplement: Supplementary file 1 — cs1c02150_si_001.pdf [file cs1c02150_si_001.pdf]

SUPPLEMENTARY INFORMATION

A Combined Theoretical and Experimental Study to Unravel the Differences in Promiscuous Amidase Activity of Two Nonhomologous Enzymes

Miquel À. Galmés,[1] Alexander R Nödling,[2] Louis Luk,[2,*] Katarzyna Świderek,[1,*] Vicent Moliner[1,*]

1. Institute of Advanced Materials (INAM), 12071 Castellón, Spain.
2. School of Chemistry, Cardiff University, Main Building, Park Pl, Cardiff CF10 3AT, UK.

corresponding authors:

L. Luk: lukly@cardiff.ac.uk; K. Świderek: swiderek@uji.es; V. Moliner: moliner@uji.es

RESULTS AND ANALYSIS

```
Bs2     1 MTHQIVTTQYGKVKGTTENGVHKWKGIPYAKPPVGQWRFKAPEPPEVWEDVLDATAYGSICPQPSDLLSLSYTELPRQSEDCLYVNVFAPDTPSKNLPVM  100
           ||||||||||||||||||||||||||||||||||||||||||||||||||||||||||.||||||         ||||||||||||||||||||:|||||
1QE3    2 -THQIVTTQYGKVKGTTENGVHKWKGIPYAKPPVGQWRFKAPEPPEVWEDVLDATAYGPICPQPS---------LPRQSEDCLYVNVFAPDTPSQNLPVM  100

Bs2   101 VWIHGGAFYLGAGSEPLYDGSKLAAQGEVIVVTLNYRLGPFGFLHLSSFNEAYSDNLGLLDQAAALKWVRENISAFGGDPDNVTVFGESAGGMSIAALLA  200
          |||||||||||||||||||||||||||||||||||||||||||||||||:||||||||||||||||||||||||||||||||||||||||||||||||||
1QE3  101 VWIHGGAFYLGAGSEPLYDGSKLAAQGEVIVVTLNYRLGPFGFLHLSSFDEAYSDNLGLLDQAAALKWVRENISAFGGDPDNVTVFGESAGGMSIAALLA  200

Bs2   201 MPAAKGLFQKAIMESGASRTMTKEQAASTSAAFLQVLGINEGQLDKLHTVSAEDLLKAADQLRIAEKENIFQLFFQPALDPKTLPEEPEKAIAEGAASGI  300
          |||||||||||||||||||||||||||||:|||||||||||.|||:||||:|||||||||||||||||||||||||||||||||||||||:|||||||||
1QE3  201 MPAAKGLFQKAIMESGASRTMTKEQAASTAAAFLQVLGINESQLDRLHTVAAEDLLKAADQLRIAEKENIFQLFFQPALDPKTLPEEPEKSIAEGAASGI  300

Bs2   301 PLLIGTTRDEGYLFFTPDSDVHSQETLDAALEYLLGKPLAEKVADLYPRSLESQIHMMTDLLFWRPAVAYASAQSHYAPVWMYRFDWHPKKPPYNKAFHA  400
          ||||||||||||||||||||||||||||||||||||||||||.||||||||||||||||||||||||||||||||||||||||||||||:||||||||||
1QE3  301 PLLIGTTRDEGYLFFTPDSDVHSQETLDAALEYLLGKPLAEKAADLYPRSLESQIHMMTDLLFWRPAVAYASAQSHYAPVWMYRFDWHPEKPPYNKAFHA  400

Bs2   401 LELPFVFGNLDGLERMAKAEITDEVKQLSHTIQSAWITFAKTGNPSTEAVNWPAYHEETRETLILDSEITIENDPESEKRQKLFPSKGE  489
          |||||||||||||       ||||||||||||||||||||||||||||||||||||||||||:|||||||||||||||||||||
1QE3  401 LELPFVFGNLDGL-------ITDEVKQLSHTIQSAWITFAKTGNPSTEAVNWPAYHEETRETVILDSEITIENDPESEKRQKLF-----  484
```

Figure S1. Sequence alignment between Bs2 available crystal structure (PDB ID: 1QE3, NRRL B8079 strain) and sequence of Bs2 from strain 168. In red the residues of catalytic triad are highlighted. In yellow missing fragments of protein, and in green the differences in sequence between both models are indicated.

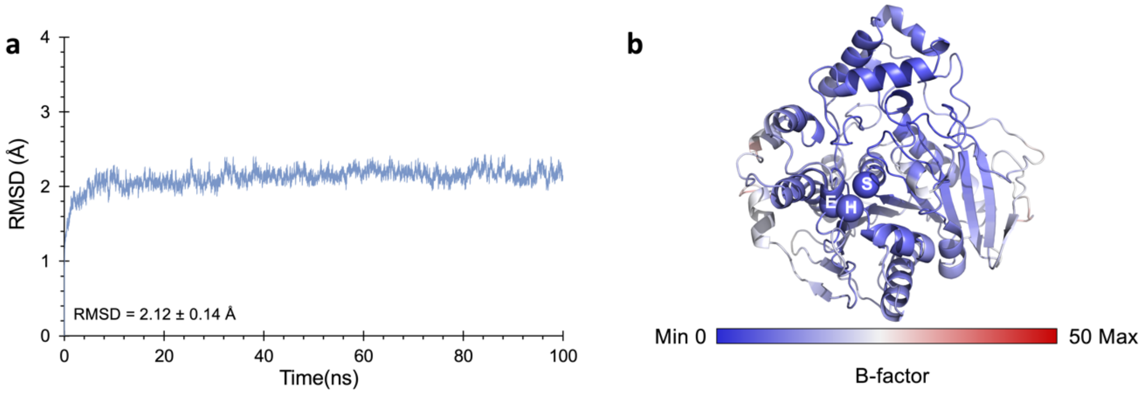

Figure S2. Analysis of 100 ns molecular dynamic simulations. **a:** Root mean square deviation (RMSD) of the position of Cα, C, and N atoms of the backbone along 100ns of MD simulation. **b:** Representation of the computed B-factor for Bs2. The catalytic triad is highlighted as spheres.

```
Bs2    1 MTHQIVTTQYGKVKGTTENGVHKWKGIPYAKPPVGQWRFKAPE-------------PPEVWEDVLDATAYGSICPQPSD------LLSLSYTEL-----P  76
                                   :|....|.                      |..|.:.:|.....|:..||..|      ...|.||..     |
CALB   1 --------------------------LPSGSDPA----FSQPKSVLDAGLTCQGASPSSVSKPILLVPGTGTTGPQSFDSNWIPLSTQLGYTPCWISPPP  70

Bs2   77 RQSED-------------CLYVNVFAPDTPSKNLPVMVWIHGG-------AFYLGAGSE--------PLYDGSKLAAQGEVIVVTLNYRLGPFGFLHLSS 148
         ....|             .||..     :.:..|||:.|..||       .|:....|:        |.|.|:.||              ||...|.:|
CALB  71 FMLNDTQVNTEYMVNAITALYAG-----SGNNKLPVLTWSQGGLVAQWGLTFFPSIRSKVDRLMAFAPDYKGTVLA--------------GPLDALAVS- 150

Bs2  149 FNEAYSDNLGLLDQAAALKWVRENISAFGGDPDNVTVFGESAGGMS--IAALLAMPAAKGLFQKAIMESGASRT-----------------MTKEQAAST 229
                        |...|.:...||       :|....:|||::  :.......|...:.|..:..|....:                 ...:.|.|.
CALB 151 ---------------APSVWQQTTGSA-------LTTALRNAGGLTQIVPTTNLYSATDEIVQPQVSNSPLDSSYLFNGKNVQAQAVCGPLFVIDHAGSL 228

Bs2  230 SAAFLQVLG-----------------------INEGQLDKLHTVSAEDLL-KAADQLRIAEKENIFQLFFQPALDPKTLPEEPEKAIAEGAASGI--PLL 303
         ::.|..|:|                       :....|.....|:|..|| .||..:....|:|         .:|..:|.....|:.:...|||  |
CALB 229 TSQFSYVVGRSALRSTTGQARSADYGITDCNPLPANDLTPEQKVAAAALLAPAAAAIVAGPKQN---------CEPDLMPYARPFAVGKRTCSGIVTP-- 317

Bs2  304 IGTTRDEGYLFFTPDSDVHSQETLDAALEYLLGKPLAEKVADLYPRSLESQIHMMTDLLFWRPAVAYASAQSHYAPVWMYRFDWHPKKPPYNKAFHALEL 403

CALB 317 ---------------------------------------------------------------------------------------------------- 317

Bs2  404 PFVFGNLDGLERMAKAEITDEVKQLSHTIQSAWITFAKTGNPSTEAVNWPAYHEETRETLILDSEITIENDPESEKRQKLFPSKGE 489

CALB 317 -------------------------------------------------------------------------------------- 317
```

Figure S3. Sequence alignment between CALB and Bs2. The alignment was done using the Needleman-Wunsch[1] algorithm implemented in EMBOSS-6.6.0[2] package (scoring matrix: BLOSUM62;[3] gap penalty: 10; gap extension penalty: 0.5). Residues of catalytic triad are highlighted in red.

Table S1. Key inter-atomic distances of the different states appearing along the hydrolysis of *N*-(4-nitrophenyl)-butyramide catalyzed by wild-type Bs2. Structures optimized at M06-2X/MM level. All distances are given in Å.

	RC	TS1	INT1	INT1	TS2	INT2	INT2	TS3	INT3	INT3	TS4	PC
$OG_{Ser189} – C1_{subs}$	2.32	1.80	1.49	1.48	1.35	1.33	1.33	1.40	1.46	1.44	1.71	2.35
$OG_{Ser189} – HG_{Ser189}$	1.00	1.22	2.08	2.44	2.71	2.58	-	-	-	-	-	-
$NE2_{His399} – HG_{Ser189}$	1.69	1.32	1.03	1.03	1.04	1.76	-	-	-	-	-	-
$C1_{subs} – N4_{subs}$	1.37	1.44	1.53	1.55	2.26	2.62	-	-	-	-	-	-
$HG_{Ser189} – N4_{subs}$	2.76	2.62	2.25	2.05	1.96	1.05	-	-	-	-	-	-
$O_{wat} – C1_{subs}$	-	-	-	-	-	-	2.53	1.69	1.49	1.48	1.41	1.34
$O_{wat} – H1_{wat}$	-	-	-	-	-	-	0.98	1.19	1.74	1.98	2.44	2.69
$H1_{wat} – NE2_{His399}$	-	-	-	-	-	-	1.91	1.34	1.05	1.03	1.30	1.74
$H1_{wat} – OG_{Ser189}$	-	-	-	-	-	-	2.88	2.52	2.60	2.76	1.31	1.00
$HD1_{His399} – OE1_{Glu310}$	1.64	1.60	1.50	1.55	1.55	1.68	1.77	1.72	1.62	1.69	1.54	1.75
$HD1_{His399} – ND1_{His399}$	1.04	1.04	1.07	1.07	1.07	1.03	1.03	1.04	1.05	1.04	1.06	1.03
$C1_{subs} – O2_{subs}$	1.24	1.27	1.31	1.31	1.24	1.23	1.23	1.27	1.31	1.31	1.27	1.23
$O2_{subs} – H_{Ala190}$	1.93	1.85	1.79	1.87	1.81	1.79	1.77	1.93	1.87	1.76	1.72	1.96
$O2_{subs} – H_{Ala107}$	1.80	1.87	1.86	1.89	1.94	1.93	1.98	1.93	1.90	1.74	1.90	1.72

Table S2. CHelpG charges (in a.u.) of the key atoms of the amide substrate computed at M06-2X/MM level on the states involved in the full chemical reaction catalyzed by the wild-type Bs2 and CALB (data from ref 11) from reactants complex (RC) to products complex (PC).

Bs2

	$C1_{subs}$	$N4_{subs}$	$O2_{subs}$	$NE2_{His399}$	OG_{Ser189}	O_{wat}
RC	0.794	-0.865	-0.846	-0.268	-0.554	
TS1	0.790	-0.850	-0.947	-0.103	-0.513	-
INT1	0.823	-0.826	-1.051	-0.142	-0.572	-
INT1	0.790	-0.822	-1.040	-0.156	-0.542	-
TS2	0.768	-0.906	-0.842	-0.187	-0.482	-
INT2	0.696	-0.927	-0.793	-0.396	-0.423	-
INT2	0.901	-	-0.810	-0.327	-0.340	-0.829
TS3	0.909	-	-0.965	-0.183	-0.455	-0.687
INT3	0.942	-	-1.064	-0.118	-0.502	-0.737
INT3	0.818	-	-1.061	-0.213	-0.494	-0.935
TS4	1.013	-	-0.987	-0.142	-0.536	-0.825
PC	0.747	-	-0.805	-0.256	-0.536	-0.673

CALB

	C1	O2	N4
RS	0.85 ± 0.03	-0.884 ± 0.012	-0.77 ± 0.03
TS1	0.92 ± 0.04	-1.009 ± 0.020	-0.888 ± 0.023
INT1	1.04 ± 0.03	-1.104 ± 0.017	-0.89 ± 0.03
TS2	0.85 ± 0.04	-1.086 ± 0.015	-0.49 ± 0.03
INT2	0.82 ± 0.03	-0.806 ± 0.013	-0.78 ± 0.04
TS3	0.74 ± 0.04	-0.965 ± 0.022	-
INT3	0.73 ± 0.03	-1.039 ± 0.015	-
TS4	0.96 ± 0.04	-1.030 ± 0.021	-
PS	0.76 ± 0.03	-0.848 ± 0.013	-

Table S3. Electrostatic potential (in kJ·mol^{-1}·e^{-1}) generated by the enzyme on the key atoms of the reaction along the states appearing in the acylation step in Bs2 and CALB.

	Bs2					CALB				
	C1	N4	O2	OG	NE2	C1	N4	O2	OG	NE2
RC	386.8	287.8	525.2	380.5	285.4	-10.9	-85.2	150.1	-68.3	-134.5
TS1	387.4	289.1	528.1	373.9	288.9	-3.4	-76.4	169.0	-58.3	-131.0
I1	393.8	290.5	536.9	385.5	280.3	-0.3	-73.5	178.3	-52.7	-132.7
TS2	384.9	249.5	509.4	369.3	246.7	-13.5	-89.6	150.6	-47.0	-131.5
I2	410.9	250.7	519.8	395.4	271.4	-11.2	-118.6	142.5	-55.8	-142.6

Table S4. Projection of the electric field created by the proteins Bs2 and CALB in the C−N peptide bond direction, computed in C1 and N4 atoms, and the resulting electrostatic forces on the direction of the vector defined from C1 to N4 atoms. All values are in a.u.

	Bs2				CALB			
	Electric Field		Force		Electric Field		Force	
	C1	N4	C1	N4	C1	N4	C1	N4
RC	0.0138	0.0135	0.0110	-0.0117	0.0119	0.0095	0.0101	-0.0073
TS1	0.0129	0.0132	0.0102	-0.0112	0.0102	0.0096	0.0094	-0.0085
INT1	0.0134	0.0129	0.0110	-0.0107	0.0097	0.0096	0.0101	-0.0085
TS2	0.0110	0.0110	0.0084	-0.0100	0.0104	0.0076	0.0089	-0.0037
INT2	0.0128	0.0102	0.0089	-0.0095	0.007	0.0062	0.0057	-0.0048

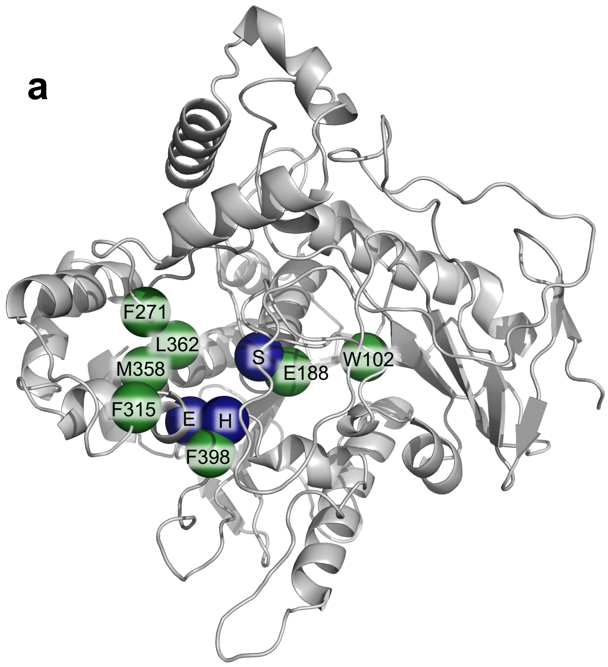

Figure S4. Representation of the key residues identified by the alanine scan using the CNN on both Bs2 (a) and CALB (b). Spheres correspond to the CA of each residue. In blue is marked the catalytic triad while in green the key residues are highlighted.

COMPUTATIONAL METHODS

Models set up. Wild type *p*-nitrobenzyl (PNB) esterase sequence from *Bacillus subtilis* (with ID P37967) that corresponds to the protein used in the experimental measurements[4] was initially taken from UniProt[5] accessible resource of protein sequence and functional information. Due to the lack of crystallized structure for this specific variant, the framework for the required model, a natural variant of the protein (strain 168), was prepared based on PNB esterase from the same organism (Bs2; PDB ID: 1QE3).[6] Both enzymes share 97.6 % of sequence identity. Thus, the X-ray structure was used as a template to build the Bs2 model by adding the missing residues and introducing required mutations. All changes were done using Modeller.[7] The differences in sequence together with the regions of missing residues are presented in Figure S1.

N-(4-nitrophenyl)-butyramide substrate was placed inside the active site pocket covalently bound to catalytic Ser189 in the form of intermediate 1, in order to avoid possible substrate dissociation to the solvent during MD simulations. The protonation state of titratable residues was determined at pH 7 by estimating pKa shifts generated by the local environment on titratable groups using the empirical program PropKa ver. 3.0.3.[8,9] The values of pKa obtained for Glu188 (8.72), Glu214 (7.52), and Glu402 (10.32) ensured their protonated state. Additionally, all histidine residues present in the enzyme were found to be neutral, with pKa values varying between 4.12 and 6.19. After a detailed inspection of surrounding of each histidine residue, it was concluded that all should be protonated in Nδ position. Thus, once the hydrogen atoms were added to the structure, the 20 counterions ions were placed in the most electrostatically favorable positions in order to neutralize the system. Subsequently, the system was solvated by placing it in a 100 × 80 × 80 Å^3 pre-equilibrated box of TIP3P[10] water molecules. Any water with an oxygen atom lying in a radius of 2.8 Å from a heavy atom of the

protein was deleted. In order to equilibrate the total system, classical MD simulations were done. For *N*-(4-nitrophenyl)-butyramide the same force field parameters were used as determined in our previous work.[11] Such prepared model was optimized, then the system was heated to 303 K with 0.1 K temperature increment and equilibrated during short (100 ps) NPT MD simulations, the proper 100 ns of non-accelerated classical MD simulations were done using the NVT ensemble with AMBER force field,[12] as implemented in NAMD software.[13] The temperature during the MD simulation was controlled using the Langevin thermostat.[14] In order to improve the time of simulations, cut-offs for nonbonding interactions were applied using a smooth switching function between 14.5 and 16 Å. During MD simulations all atoms were free to move. Periodic boundary conditions were used. Time-dependent evolution of the root mean square deviations (RMDS) together with B-factor are depicted in Figure S2. Thus, one structure from the MD simulation was chosen for further study applying QM/MM approach. The starting structures were selected by its proximity to the average values of RMSD.

QM/MM simulations. In present work, the standard additive hybrid QM/MM scheme was used to construct the total Hamiltonian, $\widehat{\mathrm{H}}_{\mathrm{QM/MM}}$, where the total energy $E_{QM/MM}$ is obtained as the sum of specific contributions, as presented in equation 1:

$$E_{QM/MM} = \left\langle \Psi \middle| \widehat{H_o} \middle| \Psi \right\rangle + \left(\sum \left\langle \Psi \middle| \frac{q_{MM}}{r_{e,MM}} \middle| \Psi \right\rangle + \sum\sum \frac{Z_{QM} q_{MM}}{r_{QM,MM}} \right) + E_{QM/MM}^{vdW} + E_{MM} \quad (1)$$

where E_{MM} is the energy of the MM subsystem term, $E_{QM-MM}^{vdW}$the van der Waals interaction energy between the QM and MM subsystems and E_{QM-MM}^{elect} includes both the Coulombic interaction of the QM nuclei (Z_{QM}) and the electrostatic interaction of the polarized electronic wave function (q_{MM}) with the charges of the protein (q_{MM}). The region described by quantum

mechanics includes the side chains of the catalytic Ser189, His399 and Glu310 residues as well as the full substrate, and one water molecule, as shown in Figure 2 of the main text. Three-link atoms[15] were inserted where the QM/MM boundary intersected covalent bonds: these were placed between the Ca-Cb for Ser189, His299 and Glu310. Finally, in QM part the 55 and 42 atoms were defined including link atoms, for acylation and deacylation process, respectively. The lower number of atoms included in the QM region for the deacylation step is a consequence of the fact that the first product of the studied reaction is leaving the active site in the step preceding this deacylation process. The rest of the protein, counterions and solvent molecules (in total 73077 atoms) were represented by classical OPLS-AA force field[16] and TIP3P force fields, respectively, as implemented in fDynamo library.[17] The Austin Model 1 (AM1)[18] semiempirical Hamiltonian and the Minnesota Functional M06-2X,[19] with the standard 6-31+G(d,p) basis set, were used to treat the QM sub-set of atoms, as implemented in Mopac[20] and Gaussian 09,[21] respectively. The atom positions of all residues presented beyond 25 Å from the substrate were frozen and the same cut-offs as in MD simulations were applied for the nonbonding interactions.

Potential Energy Surfaces. Potential Energy Surfaces (PES) were explored by choosing and scanning the appropriate combination of internal coordinates (ξ_i) assuming their dominant role in the shape of the reaction coordinate. Thus, a combination of different distances was controlled during exploration of all four chemical steps being part of the complete reaction path. In the first step of the reaction, the PES was generated by controlling the distance between nitrogen, NE2 atom of His399 and hydrogen, HG atoms of Ser189, together with the distance between oxygen, OG atom of Ser189 and carbonyl carbon, C1 atom of a substrate, directing acylation process. In the second step antisymmetric combination of nitrogen, NE2 atom and hydrogen HG, attached to His399 and this hydrogen atom and its acceptor, nitrogen, N4 atom of the substrate, together with carbon-nitrogen (C1-N4) bond of a substrate were controlled.

PES of the third step that starts the process of deacylation was generated by scanning the antisymmetric combination of distance between oxygen, O^{wat} atom of the water molecule and hydrogen, H^{wat} of the same molecule and the same hydrogen atom and nitrogen, NE2 atom of His399, together with a distance corresponding to the formation of the covalent bond between oxygen, O^{wat} from water and carbon, C1 atom of the substrate. The final, fourth step of reaction is explored controlling the antisymmetric combination of distance between nitrogen, NE2 atom of His399 and hydrogen, H^{wat} atom and the same hydrogen atom and oxygen, OG atom of the Ser189, together with elongation distance between oxygen, OG atom of Ser 189 and carbon, C1 atom of the substrate.

In order to explore all PESs, the harmonic constraint of 5000 $kJ \cdot mol^{-1} \cdot Å^{-2}$ was used to maintain the proper interatomic distances along the reaction coordinate, and a series of conjugate gradient optimizations and L-BFGS-B optimization algorithms[22] were applied to obtain the final potential energy of the minimized constrained geometry. The QM sub-set of atoms were described by the Austin Model 1 (AM1) semiempirical Hamiltonian. The distances evolution was controlled by applying small size change of 0.1 Å when the distance between two heavy atoms was explored, or 0.05 Å when the transfer of light hydrogen atom was involved.

A micro-macro iteration optimization algorithm[23,24] together with the Baker's algorithm[25,26] was used to localize, optimize, and characterize the transition states (TS) and structures using a Hessian matrix containing all the coordinates of the QM subsystem, whereas the gradient norm of the remaining movable atoms was maintained less than 0.25 $kcal\ mol^{-1}\ Å^{-1}$. The Intrinsic Reaction Coordinate[10] (IRC) was traced down from located TSs to the connecting valleys in mass-weighted Cartesian coordinates. And the same micro-macro iteration optimization algorithm was used to optimized reactant complex (RC), intermediates (Is) and product complex (PC). The existence of the saddle-points, as well as those located in minima, was confirmed by frequency calculation. Thus, for TS structures, only one imaginary value of

frequency was registered, while for structures from located in the minimum of the PESs no imaginary values were found.

Free Energy Surfaces. FESs were obtained, in terms of two-dimensional potential mean force (2D-PMF),[27] for every step of the reaction using the Umbrella Sampling approach[27,28] combined with the Weighted Histogram Analysis Method (WHAM).[29] The procedure for the PMF calculation is straightforward and requires a series of molecular dynamics simulations in which the distinguished reaction coordinate variable, ξ, is constrained around particular values. The values of the variables sampled during the simulations are then pieced together to construct a distribution function from which the PMF is obtained as a function of the distinguished reaction coordinate (W(ξ)). The PMF is related to the normalized probability of finding the system at a particular value of the chosen coordinate by eq 2:

$$W(\xi) = C - kTln \int \rho(r^N)\delta(\xi(r^N) - \xi)dr^{N-1} \quad (2)$$

The activation free energy can be then expressed as:

$$\Delta G^{\ddagger}(\xi) = W(\xi^{\ddagger}) - \left[W(\xi^R) + G_{\xi}(\xi^R)\right] \quad (3)$$

where the superscripts indicate the value of the reaction coordinate at the reactants (R), and at the TS (‡), and $G_{\xi}(\xi^R)$ is the free energy associated with setting the reaction coordinate to a specific value at the reactant state. Normally this last term makes a small contribution, and the activation free energy is directly estimated from the PMF change between the maximum of the profile and the reactant's minimum:

$$\Delta G^{\ddagger}(\xi) \approx W(\xi^{\ddagger}) - W(\xi^R) = \Delta W^{\ddagger}(\xi) \quad (4)$$

The selection of the reaction coordinate is usually trivial when the mechanism can be driven by a single internal coordinate or a simple combination (as the antisymmetric combination of two interatomic distances). However, this is not the case for all possible steps of the reaction subject of study in this paper where many coordinates are participating. Instead, we were

compelled to obtain a much more computationally demanding 2D-PMF using two coordinates: ξ_1 and ξ_2. The 2D-PMF is related to the probability of finding the system at particular values of these two coordinates:

$$W(\xi) = C' - kTln \int \rho(r^N)\delta(\xi_1(r^N) - \xi_1)\delta(\xi_2(r^N) - \xi_2)dr^{N-2} \quad (5)$$

To estimate the activation free energy from this quantity, we recovered one-dimensional PMF changes tracing a maximum probability reaction path on the 2D-PMF surface and integrating over the perpendicular coordinate.

Thus, a series of MD simulations were performed adding a constraint for the selected reaction coordinates with an umbrella force constant of 2500 kJ·mol^{-1}·Å^{-2}. In every window, QM/MM MD simulations were performed with a total of 5 ps of equilibration and 20 ps of production at 303 K using the Langevin-Verlet algorithm[30] with a time step of 1 fs. Structures obtained in previously computed PESs were used as starting points for the MD simulations in every window. The total number of calculations for each surface is summarized in Table S5.

Table S5. The total amount of calculations required to produce each AM1/MM 2D-PMF surface corrected at M06-2X:AM1/MM level of theory.

	**Step 1**	**Step 2**	**Step 3**	**Step 4**	**TOTAL**
Geometry optimization (AM1/MM)	403	1659	976	854	3892
QM/MM MD simulation (AM1/MM)	403	1659	976	854	3892
Single Point Energy calculations (M06-2X/MM)	403	1659	976	854	3892
**TOTAL**	1209	4977	2928	2562	**11676**

Spline corrections. In order to improve lower quality results associated with the low-level semiempirical calculations, high-level corrections were applied using Density Functional Theory (DFT). As already described in the literature,[31,32] a correction term $S[\Delta E_{LL}^{HL}(\xi_1, \xi_2)]$ is interpolated to any value along reaction coordinates in the FES. A continuous energy function is used to obtain the corrected PMFs:

$$E = E_{LL/MM} + S[\Delta E_{LL}^{HL}(\xi_1, \xi_2)] \quad (6)$$

where S is the two-dimensional spline function and ΔE_{LL}^{HL} is the difference between the energies obtained at low-level (LL) and high-level (HL) of the theory of the QM part. The AM1 semiempirical Hamiltonian was used as LL method, while the DFT method was selected for the HL energy calculation. In particular, HL energy calculations were performed by means of the hybrid M06-2X functional using the standard 6-31+G(d,p) basis set. These calculations were carried out using the Gaussian09 program.

Phylogeny. The phylogenetic tree was build using a random sample of 150 sequences of proteins belonging to EC 3.1.1.1, EC 3.1.1.3 and EC 3.1.1.4. Fasta sequences were taken from Uniprot[33] database. Multiple sequence alignment was done using Clustal Omega[34]. Then the tree was constructed using BioPython[35] library. Distances were calculated using BLOSUM62[3] substitution matrix and the tree was constructed with the UPGMA[36] clustering method. The final tree was plotted using the GraPhlAn[37] package.

3D Convolutional Neural Network. Crystallographic data of enzymes belonging to EC 3.1.1.1, EC 3.1.1.3 and EC 3.1.1.4 was taken from PDB[38] database. These proteins were selected based on its classification in Uniprot[33] database. In order to avoid overrepresentation of some structures, redundancy was cleaned based on sequence alignment between all the set of proteins. All proteins that shared more than 85 % of sequence identity were eliminated. The alignment was done using the Needleman-Wunch[1] algorithm. A final list of the codes used to build the dataset is showed in Table S6. The catalytic site of each protein was then labeled based on the curated information available in Uniprot. Then, a cube of 20 × 20 × 20 Å^3 was centered on the geometrical center of the catalytic site. Placing the cube in the catalytic site permits the analysis of only the near vicinity of the active site. The cube was then divided into voxels of 1 Å to ensure that only one atom lay inside each voxel. Subsequently, all atoms that

lay inside the cube were selected and codified into a 13 vector. Each position of the vector represented a specific characteristic of the codified residue or atom, i.e., atom type (C, N, O or S), atom belonging to the backbone, methyl group, negative or positive charge, polar or non-polar, hydrogen bond donor or acceptor, or aromatic residue. If the characteristic was present in the atom the corresponding position of the vector was set to 1, and a 0 otherwise. A final cube of dimension $20 \times 20 \times 20$ $Å^3$ and 13 channels was obtained for each protein of the initial dataset. In order to mimic the electron density in a real protein, a series of Gaussian filters were applied in each channel. These filters were calibrated to resemble de Van der Waals radius of each atom type. This process was done using Python language and NumPy[39] and SciPy[40] libraries.

Table S6. PDB IDs of the proteins used for the CNN training.

EC number	PDB ID
EC 3.1.1.1	2O7V, 5FV4, 1K4Y, 1AUR, 3P2M, 5THM, 3KVN, 1TQH, 4JGG, 2R11, 4CCY
EC 3.1.1.3	5CUR, 3RAR, 1THG, 1GZ7, 1THG, 1LLF, 3GUU, 1K8Q, 1HLG, 1HPL, 1N8S, 1ETH, 1GPL, 2PVS, 1BU8, 4FKB, 5LIP, 2ES4, 1EX9, 6QPR, 1LGY, 6XS3
EC 3.1.1.4	1QD6, 4BP2, 5IZR, 1TC8, 1OZ6, 1POB, 1GP7, 1M8T, 1P7O, 4RFP, 1PP2, 1IJL, 1VAP, 1BUN, 1OO1, 6AL3, 5TFV, 2NOT, 5VET, 3G8G, 4HG9, 1PO8, 1VIP, 1RGB, 2GHN,

In order to up-sample the dataset a series of random rotations were applied to the cubes in x, y and z axis. Due to the different number of proteins in each of the EC classes, the dataset was balanced by upsampling each class independently to obtain an equal number of samples per class in the dataset. The network was trained with a ratio train:test:validation of 0.7:0.15:0.15. In this case, we are not talking about a specific number of samples in each dataset because the

cubes and the random rotations were done on the fly while the network was trained so each mini batch was in this sense different. For that reason, we trained until convergency of the loss. It must be said that our aim is not to obtain a general classifier, which would require a much bigger and diverse protein set, but a tool that permits the analysis of Bs2 and CALB. For that reason, structures of Bs2 and CALB were not used in the CNN training.

A ResNet50[41] architecture was chosen to build the CNN. In this case a set of 3D convolutional layers and 3D max pooling layers were applied maintaining the original architecture of the network. For the training of the CNN, the Adam[42] optimizer was used and the categorical crossentropy was chosen a loss function. A mini batch of 16 was set for each iteration, and an exponential decay learning rate was scheduled (starting at 10^{-4}). The CNN was build using Keras v.2.4.3 [43] and Tensorflow v.2.4.1.[44]

In order to validate the CNN for our particular task, 1000 samples applying random rotations of Bs2 and CALB were predicted, and the ratio of correct classifications was used as measure of the performance of the CNN.

Finally, an alanine scan was done in order to figure out which are the structural determinants in both Bs2 and CALB. For that, all the residues that had an atom inside the cube were mutated to alanine consecutively, obtaining a library of mutants. The mutations were introduced using Modeller.[7] Then, 1000 predictions were done in the same way as explained before. With this strategy we were able to highlight which are the residues that the CNN has learned to be crucial for its correct classification. A classification score was computed by the difference between the classification ratio of the actual class and the ratio of the most probable of the remaining classes (equation 7).

$$s = \frac{c_{i1}}{t_{i1}} - \frac{c_{\max(i2,i3)}}{t_{\max(i2,i3)}} \tag{7}$$

In the above equation *s* stands for the score, *c* is the number of correctly classified instances, *t* is the total number of instances, *i1* stands for the actual class and *i2* and *i3* are the rest of the classes. In this sense a score of 1 corresponds to a perfectly classified mutant and a score of -1 is the one completely misclassified. Finally, after each iteration the mutation that dropped the score the most was accumulated for the next iteration. In this way we performed up to 5 iterations.

STRUCTURES COORDINATES

Table S7. X, Y and Z coordinates of QM atoms for Transition State Structures optimized at M06-2X/MM level for acylation step of the reaction of hydrolysis taking place in the active site of Bs2.

Acylation step							
**Transition State 1** (ν_i = -907.500 cm^{-1})				**Transition State 2** (ν_i = -103.200 cm^{-1})			
Atoms	x	y	z	Atoms	x	y	z
C	45.471	44.021	52.769	C	45.424	43.836	52.633
H	46.369	44.586	53.017	H	46.267	44.347	53.096
H	45.012	44.525	51.912	H	45.095	44.428	51.780
O	44.583	44.140	53.888	O	44.392	43.777	53.624
H	45.068	44.609	54.901	H	44.783	45.514	55.661
C	48.614	41.110	59.894	C	48.545	41.092	59.950
H	48.447	40.206	60.490	H	48.411	40.169	60.524
H	47.796	41.151	59.168	H	47.710	41.143	59.244
C	49.925	40.969	59.125	C	49.832	40.982	59.140
H	50.803	41.082	59.768	H	50.727	41.148	59.747
H	49.968	39.959	58.706	H	49.912	39.968	58.737
C	50.101	41.910	57.939	C	49.963	41.891	57.932
O	49.231	42.789	57.701	O	49.151	42.831	57.715
O	51.104	41.728	57.191	O	50.920	41.605	57.151
C	47.478	45.820	59.213	C	47.505	45.848	59.446
H	47.161	45.209	60.063	H	47.237	45.187	60.271
H	47.113	46.835	59.398	H	47.168	46.856	59.701
C	46.789	45.274	57.965	C	46.761	45.428	58.203
N	47.002	43.974	57.581	N	47.007	44.249	57.528
H	47.748	43.340	57.945	H	47.784	43.552	57.749
C	46.346	43.741	56.448	C	46.236	44.191	56.450
H	46.373	42.798	55.924	H	46.221	43.390	55.728
N	45.670	44.812	56.056	N	45.482	45.289	56.398
C	45.939	45.792	57.006	C	45.797	46.084	57.482
H	45.520	46.785	56.933	H	45.339	47.048	57.642
C	42.965	44.864	53.581	C	43.165	44.284	53.353
C	42.155	43.939	54.483	C	42.176	43.756	54.351
H	42.620	43.909	55.475	H	42.625	43.770	55.348
H	41.175	44.422	54.612	H	41.296	44.403	54.364
C	41.971	42.540	53.924	C	41.829	42.320	53.930
C	41.008	41.711	54.765	C	40.984	41.592	54.962
H	40.026	42.197	54.820	H	40.051	42.131	55.157
H	40.866	40.716	54.333	H	40.732	40.588	54.608
H	41.371	41.594	55.791	H	41.518	41.496	55.912
H	41.601	42.614	52.894	H	41.310	42.342	52.963
H	42.947	42.048	53.863	H	42.755	41.760	53.759
O	42.813	44.812	52.321	O	42.850	44.625	52.203
N	43.052	46.149	54.233	N	43.369	46.220	54.495
H	42.399	46.277	55.018	H	42.617	46.411	55.179
C	43.757	47.256	53.858	C	43.906	47.351	54.061
C	44.609	47.292	52.731	C	44.810	47.361	52.934
C	45.345	48.423	52.439	C	45.486	48.487	52.557
C	45.255	49.543	53.273	C	45.330	49.683	53.298
C	44.403	49.549	54.387	C	44.398	49.738	54.365
C	43.656	48.423	54.662	C	43.673	48.629	54.698
H	43.001	48.407	55.530	H	42.961	48.664	55.518
H	44.332	50.431	55.020	H	44.265	50.667	54.912
N	46.066	50.696	53.001	N	46.135	50.785	53.025
O	46.685	50.767	51.945	O	46.878	50.803	52.033
O	46.130	51.588	53.856	O	46.117	51.751	53.824
H	45.996	48.423	51.571	H	46.156	48.476	51.705
H	44.677	46.432	52.084	H	44.943	46.439	52.383
H	48.478	45.821	59.198	H	48.503	45.848	59.373
H	48.571	41.899	60.507	H	48.511	41.867	60.580
H	45.730	43.098	52.486	H	45.696	42.917	52.346

Table S8. X, Y and Z coordinates of QM atoms for Transition State Structures optimized at M06-2X/MM level for deacylation step of the reaction of hydrolysis taking place in the active site of Bs2.

**Deacylation step**							
**Transition State 3** (ν_i = -646.598 cm^{-1})				**Transition State 4** (ν_i = -1212.657 cm^{-1})			
Atoms	x	y	z	Atoms	x	y	z
C	5.472	0.119	-1.252	C	52.634	45.258	40.947
H	5.977	-0.698	-1.770	H	52.915	44.309	40.494
H	4.637	0.453	-1.873	H	51.846	45.686	40.321
O	6.414	1.177	-1.069	O	53.801	46.107	40.837
H	8.058	1.197	-2.973	H	54.845	45.575	40.245
C	12.640	-2.987	1.405	C	59.844	42.390	43.238
H	13.245	-2.843	2.307	H	60.339	42.777	44.134
H	11.921	-2.163	1.376	H	59.049	43.115	43.029
C	11.854	-4.286	1.521	C	59.193	41.050	43.577
H	12.480	-5.172	1.379	H	59.893	40.210	43.544
H	11.438	-4.352	2.532	H	58.821	41.106	44.603
C	10.657	-4.418	0.586	C	57.987	40.662	42.733
O	10.403	-3.520	-0.259	O	57.718	41.297	41.677
O	9.924	-5.437	0.766	O	57.251	39.731	43.165
C	12.010	-1.891	-3.329	C	59.049	43.151	38.627
H	12.841	-1.595	-2.684	H	59.930	43.580	39.110
H	12.264	-1.576	-4.346	H	59.076	43.455	37.577
C	10.780	-1.107	-2.917	C	57.805	43.803	39.284
N	10.169	-1.247	-1.692	N	57.510	43.559	40.605
H	10.413	-1.957	-0.978	H	57.873	42.744	41.175
C	9.129	-0.404	-1.632	C	56.418	44.238	40.951
H	8.469	-0.306	-0.783	H	55.982	44.223	41.936
N	9.025	0.296	-2.754	N	55.950	44.959	39.940
C	10.052	-0.139	-3.571	C	56.821	44.692	38.876
H	10.203	0.263	-4.562	H	56.686	45.134	37.900
C	6.260	2.364	-1.801	C	53.511	47.603	40.072
C	7.156	3.395	-1.126	C	54.524	48.488	40.797
H	8.170	2.988	-1.043	H	55.488	47.968	40.848
H	7.207	4.281	-1.770	H	54.683	49.362	40.149
C	6.629	3.758	0.259	C	54.091	48.962	42.176
C	7.575	4.691	0.997	C	55.120	49.914	42.783
H	7.727	5.620	0.433	H	54.790	50.291	43.755
H	7.179	4.958	1.982	H	56.088	49.420	42.925
H	8.557	4.229	1.135	H	55.289	50.777	42.127
H	5.637	4.217	0.166	H	53.117	49.456	42.099
H	6.488	2.842	0.841	H	53.942	48.098	42.834
O	5.082	2.706	-2.148	O	52.273	47.883	40.158
O	7.168	1.959	-3.165	O	53.947	47.299	38.769
H	7.495	2.779	-3.577	H	54.749	47.829	38.530
H	11.956	-2.889	-3.305	H	59.137	42.157	38.680
H	13.245	-2.944	0.609	H	60.508	42.395	42.490
H	5.153	-0.191	-0.356	H	52.261	45.073	41.856

EXPERIMENTAL METHODS

General information. Deionized water was obtained by an *Elga PURELAB Option* system (15 MΩ·cm). Analytical Thin Layer Chromatography (TLC) was carried out with silica gel 60 F254 aluminum sheets from *Merck*. Detection was carried out using UV light (λ = 254 nm and 366 nm), followed by immersion in permanganate or cerium ammonium molybdate staining solution with subsequent development via careful heating with a heat gun. Flash column chromatography was performed using silica gel (pore size 60 Å, 0.040-0.063 mm). *N*-(4-nitrophenyl)-butyramide was synthesized using a known procedure.[11] All other solvents and reagents were obtained from commercial sources and used as received.

Plasmid miniprep-kit and gel extraction-kit were purchased from *Qiagen*. DNA oligos were purchased from *Sigma-Aldrich*. The gene encoding for wild-type Bs2[4] with a C-terminal His-tag (see below) was purchased as a double-stranded fragment form *Thermo Fisher Scientifcic GeneArt*. Gibson Assembly was performed using *New England Biolabs NEBuilder® HiFi DNA Assembly* master mix. Restriction enzymes and required reagents were obtained from *Thermo Fisher Scientific*. *Takara PrimeSTAR Max* was employed for site-directed mutagenesis. All kits and enzymes were used exactly following the maufacturers' protocols. DNA sequencing of constructed plasmids and mutants was obtained from *Eurofins Genomics* using T7 promoter and terminator primers.

A *VWR 3510* benchtop pH Meter connected to a *Jenway* micro pH electrode or a *VWR Universal* pH electrode were used for the pH adjustment of buffers and reaction mixtures employing either 1.0 M or 0.1 M sodium hydroxide solution or hydrochloric acid.

Size exclusion chromatography was performed using a *GE Healthcare ÄKTA Purifier* workstation or a *Bio-Rad NGC Medium-Pressure Liquid Chromatography System*.

Protein concentrations were determined using a *Thermo Scientific NanoDrop One* spectrophotometer measuring the absorption at 280 nm using the

Protein liquid chromatography-mass spectrometry (LC-MS) was performed on a *Waters Acquity H-Class UPLC* system combined with a *Waters Synapt G2-Si* quadrupole time of flight mass spectrometer. A *Waters Acquity UPLC Protein C4 BEH* column 300 Å, 1.7 µm (2.1 × 100 mm) held at 60 °C was applied. A flow rate of 0.2 mL/min and the gradient of eluents A and B highlighted below were employed.

**Time / min**	**A (H_2O, 0.1% CHO_2H) / %**	**B (ACN, 0.1% CHO_2H) / %**
0	95	5
3	95	5
50	35	65
52	3	97
54	3	97
56	95	5
60	95	5

The data was collected in positive electrospray ionization mode and analyzed using *Waters MassLynx 4.1*. Deconvoluted mass spectra were generate using the maximum entropy 1 (*MaxEnt 1*) software.

Cloning, expression and purification of recombinant proteins. The gene encoding for the wild-type Bs2 with a C-terminal linker and His-tag (`GSSHHHHHHSSG`), and 27 and 20 bases either side complimentary to the vector was purchased as a double-stranded fragment. After a NcoI and BamHI restriction enzyme digested pET28a vector the wild-type Bs2 gene was cloned in-between by Gibson assembly. The incorporation of the wild-type Bs2 gene was confirmed by DNA sequencing.

The plasmid containing the gene for wild-type Bs2 was transformed into Ca^{2+} chemically competent BL21 (DE3) cells and grown on LB agar plates supplemented with kanamycin (50 µg/mL) at 37 °C overnight. One colony from the plate was picked to inoculate a 10 mL LB starter culture containing kanamycin (50 µg/mL) and grown at 37 °C and 180 rpm overnight. The starter culture was diluted into 1 L of fresh LB media containing kanamycin (50 µg/mL). The cells were grown at 37 °C and 200 rpm, until they reached an OD600 of 0.8, and IPTG was added to reach a final concentration of 1.0 mM. The cells were then incubated overnight at 20 °C. The cultures were harvested by centrifugation (4,000 rpm, 4 °C, 30 min) and the dry pellet was stored at –20 °C.

The pellet was subjected to a freeze-thaw cycle, resuspended in 25 mL of lysis buffer 1 (50 mM NaP_i, 300 mM NaCl, pH 7.5) and lysed by sonication (7 min, 5 s on, 10 s off). The insoluble fraction was removed by centrifugation at 18000 rpm for 25 min at 4 °C. The supernatant was mixed with 3 mL of Ni-NTA affinity resin for His-tag affinity purification which was equilibrated with the lysis buffer. After incubation at 4 °C for 0.5 h the resin was washed twice with 1.5 volumes of resin wash buffer 1 (50 mM NaP_i, 300 mM NaCl, 10 mM imidazole, pH 7.5). The protein was eluted with elution buffer (5× resin volume, 50 mM NaP_i, 300 mM NaCl, 250 mM imidazole, pH 7.5). Samples of the wash and elution fractions were collected and run on SDS-PAGE gel (12% w/v). The elution fractions containing the respective Bs2 variant were pooled and concentrated to 5 mL using *Amicon* ultra centrifugation with a 10 kDa cut-off. The concentrated protein solution was applied to size exclusion chromatography (*Generon ProSEC 26/60 3-70 HR* column, 50 mM NaP_i, pH 7.0). Fractions containing protein (analysis by following 280 nm UV trace) were collected and the samples loaded on SDS-PAGE to check the purity of the protein (12% w/v). Fractions containing Bs2 were pooled, transferred to a centrifugal concentrator with a 10 kDa cut-off, and were concentrated to 15–20 mg/mL as determined by nanodrop measurement at 280 nm, with ε_{280} =

80330 M^{-1} cm^{-1} calculated using https://web.expasy.org/protparam/. The protein solutions were stored at 4 °C until further usage within a week. It should be noted that the solutions showed no significant loss in activity over 4 weeks (longer stability has not been monitored) and only minimal protein precipitation was observed.

Nucleotide sequence.

```
ATGACCCACCAGATTGTTACCACACAGTATGGTAAAGTGAAAGGCACCACCGAAAATGGTGT
TCATAAATGGAAAGGTATCCCGTATGCAAAACCGCCTGTTGGTCAGTGGCGTTTTAAAGCAC
CGGAACCGCCTGAAGTTTGGGAAGATGTTCTGGATGCAACCGCATATGGTAGCATTTGTCCG
CAGCCGAGCGATCTGCTGAGCCTGAGCTATACCGAACTGCCTCGTCAGAGCGAAGATTGTCT
GTATGTTAATGTTTTTGCACCGGATACGCCGAGCAAAAATCTGCCGGTTATGGTTTGGATTC
ATGGTGGTGCATTTTATCTTGGTGCAGGTAGCGAACCGCTGTATGATGGTAGCAAACTGGCA
GCACAGGGTGAAGTTATTGTTGTTACCCTGAATTATCGTCTGGGTCCGTTTGGTTTTCTGCA
TCTGAGCAGCTTTAATGAAGCCTATAGCGATAATCTGGGTCTGCTGGATCAGGCAGCAGCAC
TGAAATGGGTTCGTGAAAACATTAGCGCATTTGGTGGTGATCCGGATAATGTTACCGTTTTT
GGTGAAAGTGCCGGTGGTATGAGCATTGCAGCACTGCTGGCCATGCCTGCAGCAAAAGGTCT
GTTTCAGAAAGCAATTATGGAAAGCGGTGCAAGCCGTACCATGACCAAAGAACAGGCAGCAA
GTACCAGCGCAGCATTTCTGCAGGTTCTGGGTATTAATGAAGGTCAGCTGGATAAACTGCAT
ACCGTTAGCGCAGAAGATTTACTGAAAGCAGCAGATCAGCTGCGTATTGCAGAAAAAGAAAA
CATCTTTCAGCTGTTTTTTCAGCCTGCACTGGATCCGAAAACACTGCCGGAAGAACCGGAAA
AAGCAATTGCAGAAGGTGCAGCAAGCGGTATTCCGCTGCTGATTGGTACAACCCGTGATGAA
GGTTACCTGTTTTTTACTCCGGATAGTGATGTTCATAGCCAAGAAACCCTGGATGCAGCCCT
GGAATATCTGCTGGGTAAACCGCTGGCCGAAAAAGTTGCAGATCTGTATCCGCGTAGCCTGG
AAAGCCAGATTCATATGATGACGGATCTGCTGTTTTGGCGTCCGGCAGTTGCATATGCCAGC
GCACAGAGCCATTATGCACCGGTTTGGATGTATCGTTTTGATTGGCATCCGAAAAAACCTCC
GTATAACAAAGCATTTCATGCACTGGAACTGCCGTTTGTTTTTGGTAATCTGGATGGTCTGG
AACGTATGGCAAAAGCAGAAATTACCGATGAAGTGAAACAACTGAGCCATACCATTCAGAGC
GCATGGATTACCTTTGCAAAAACCGGTAATCCGAGCACCGAAGCAGTTAATTGGCCTGCATA
TCATGAAGAAACCCGTGAAACCCTGATTCTGGATAGCGAAATTACCATTGAAAATGATCCGG
AAAGCGAGAAACGTCAGAAACTGTTTCCGAGCAAAGGTGAAGGTAGCAGCCATCACCATCAT
CATCATAGCAGTGGTTAA
```

Protein sequence.

```
MTHQIVTTQYGKVKGTTENGVHKWKGIPYAKPPVGQWRFKAPEPPEVWEDVLDATAYGSICP
QPSDLLSLSYTELPRQSEDCLYVNVFAPDTPSKNLPVMVWIHGGAFYLGAGSEPLYDGSKLA
AQGEVIVVTLNYRLGPFGFLHLSSFNEAYSDNLGLLDQAAALKWVRENISAFGGDPDNVTVF
GESAGGMSIAALLAMPAAKGLFQKAIMESGASRTMTKEQAASTSAAFLQVLGINEGQLDKLH
TVSAEDLLKAADQLRIAEKENIFQLFFQPALDPKTLPEEPEKAIAEGAASGIPLLIGTTRDE
GYLFFTPDSDVHSQETLDAALEYLLGKPLAEKVADLYPRSLESQIHMMTDLLFWRPAVAYAS
AQSHYAPVWMYRFDWHPKKPPYNKAFHALELPFVFGNLDGLERMAKAEITDEVKQLSHTIQS
AWITFAKTGNPSTEAVNWPAYHEETRETLILDSEITIENDPESEKRQKLFPSKGEGSSHHHH
HHSSG*
```

Protein Mass. wild-type Bs2 (−Met) = 55140.21 Da

96 well-plate kinetic assay. Stock solutions of the *wild-type* Bs2 (in 50 mM NaP_i, pH 7.0) and *N*-(4-nitrophenyl)-butyramide (in DMSO) were prepared. The protein stock solution was kept on ice until use and was freshly prepared before each usage. DMSO and substrate stock solution were added to wells of a 96 transparent well-plate to a total of 15 µL. Buffer (50 mM NaP_i, pH 7.0) was added to a total volume of 135 µL (150 µL in case of controls to monitor substrate stability). The plate was transferred into a platereader, double orbitally shaken for 5 s and the absorption at λ_{Ex} = 405 nm measured to check correct substrate distribution. Then 15 µL of protein stock solution were added to each well except the enzyme free controls within 5 min. Final assay conditions were 150 µL volume, 10% DMSO, *N*-(4-nitrophenyl)-butyramide (10, 50, 100, 250 500, 1000, 2000, 3000 µM), 20 µg/mL protein. The plate was sealed with an airtight and UV-Vis transparent self-adhesive plastic cover sheet. After sealing, the plate was placed into the plate reader and the assay was monitored using the following program:

Temperature:	21.0 ± 1.0 °C
Number of repeats:	42
Delay between repeats:	1200 s
Shaking duration:	5.0 s
Shaking diameter:	0.70 mm
Shaking type:	Double orbit
Delay duration:	5.0 s
Wavelength λ_{Ex}:	405 nm

Kinetic assay analysis. In order to convert the absorption reading obtained from the kinetic assay into concentrations for the determination of kinetic data a calibration curve was generated using 10, 50, 100, 250 500, 1000, 2000, 3000 µM of 4-nitroaniline in 150 µL buffer (50 mM NaP_i, pH 7.0, 10% DMSO). Raw absorption data was converted into product concentrations and data analysis with *Origin 2020* was performed to obtained kinetic data assuming Michaelis–Menten kinetics. The maximum velocities v_{max} were obtained from the linear range of the product concentration vs. time plots and used to calculate k_{cat} and K_M for wild-type Bs2.

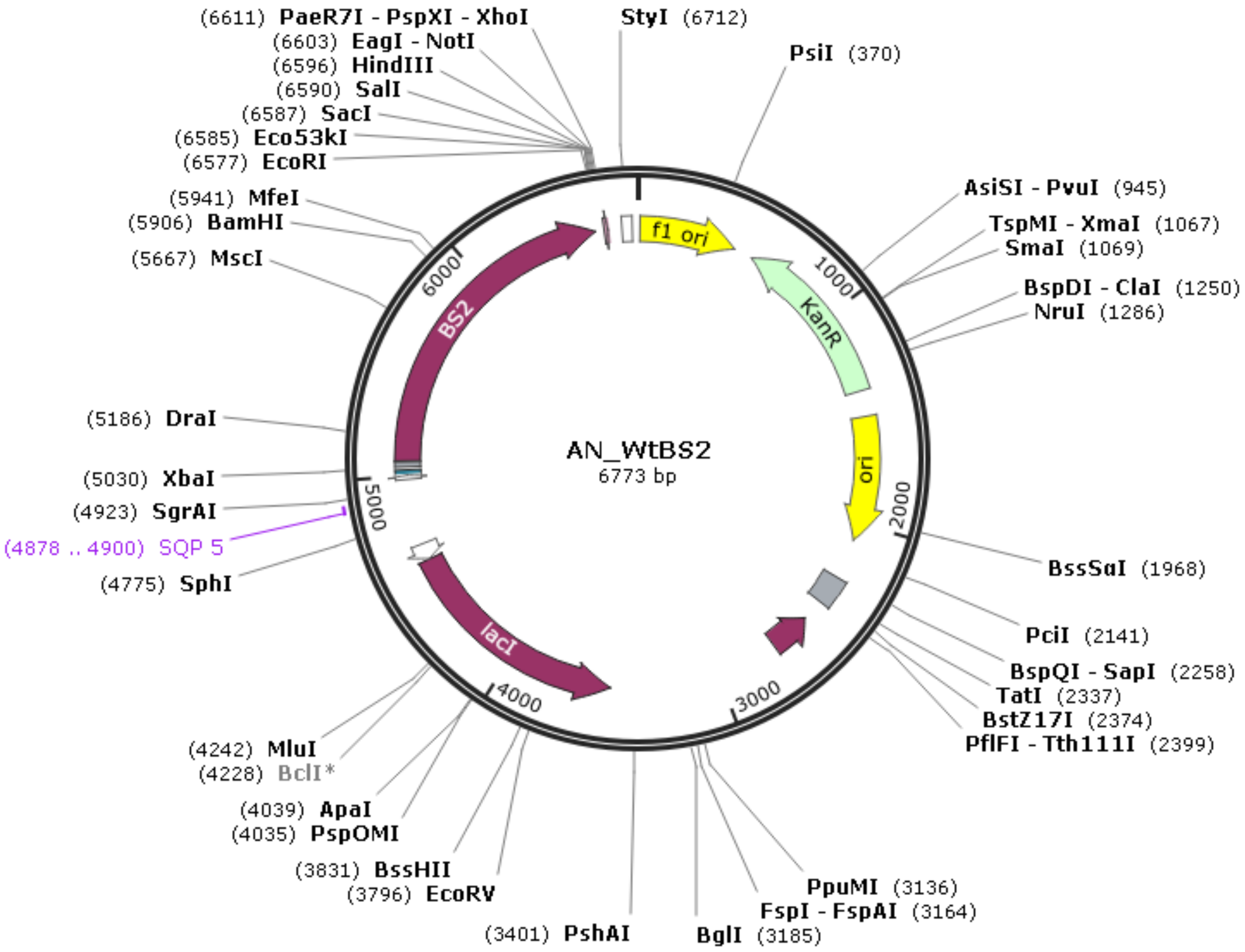

Figure S5. Plasmid map of plasmid with wild-type Bs2 gene in pET-28a vector used for recombinant protein expression.

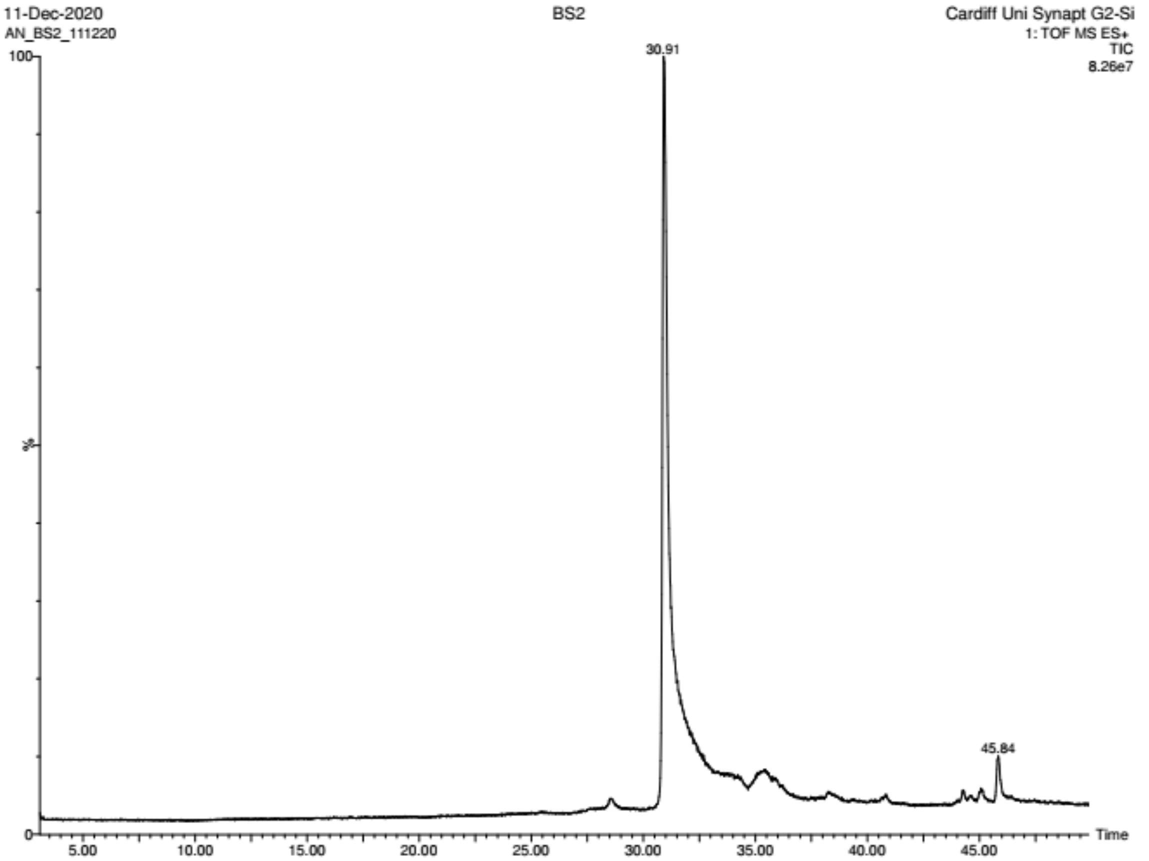

Figure S6. LC chromatogram of purified wild-type Bs2.

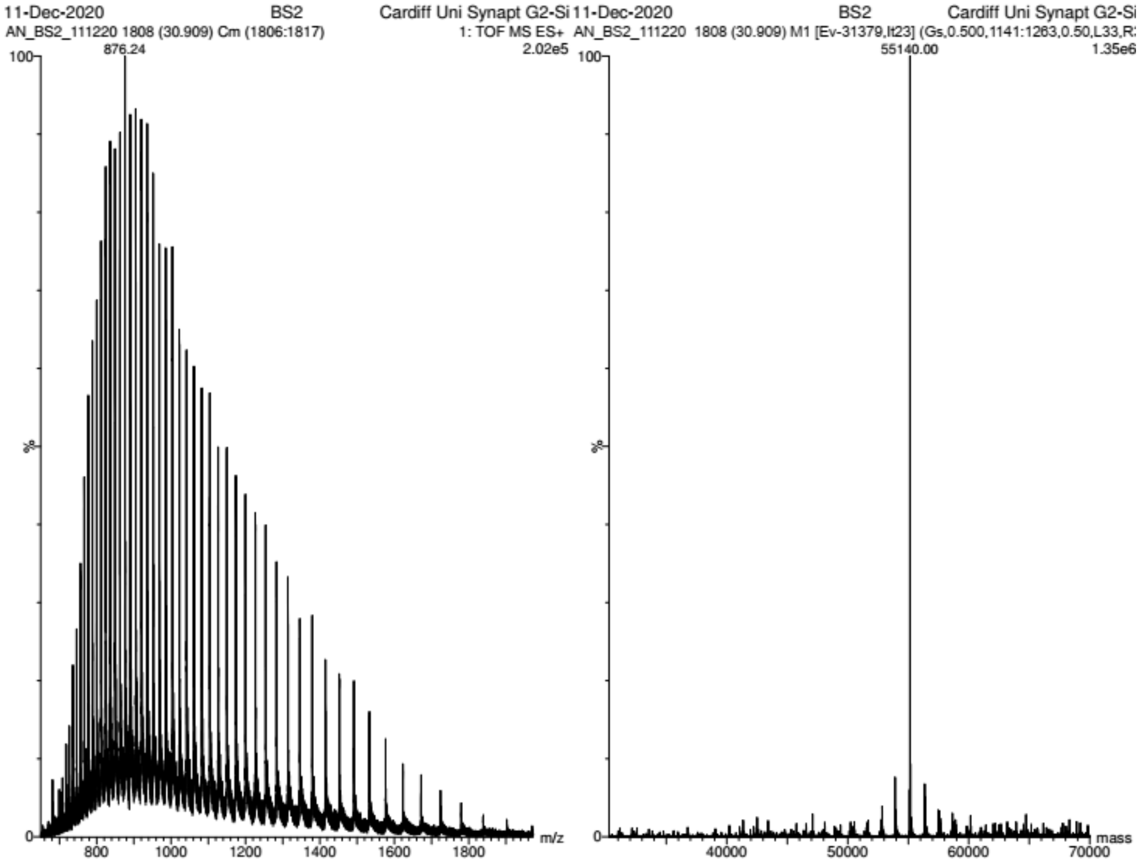

Figure S7. Raw and deconvoluted mass spectrum of purified wild-type Bs2.

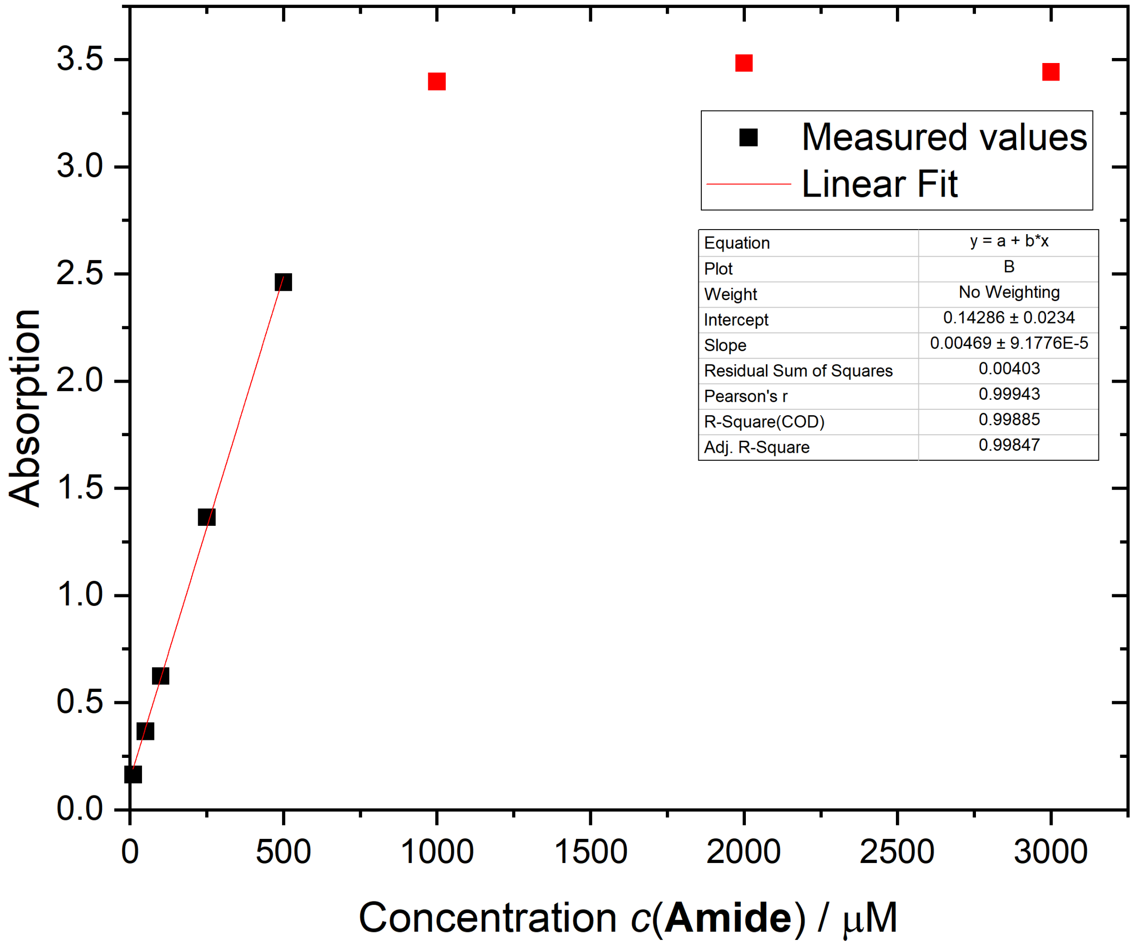

Figure S8. Calibration curve for the 4-nitroaniline concentration in the assay mixture.

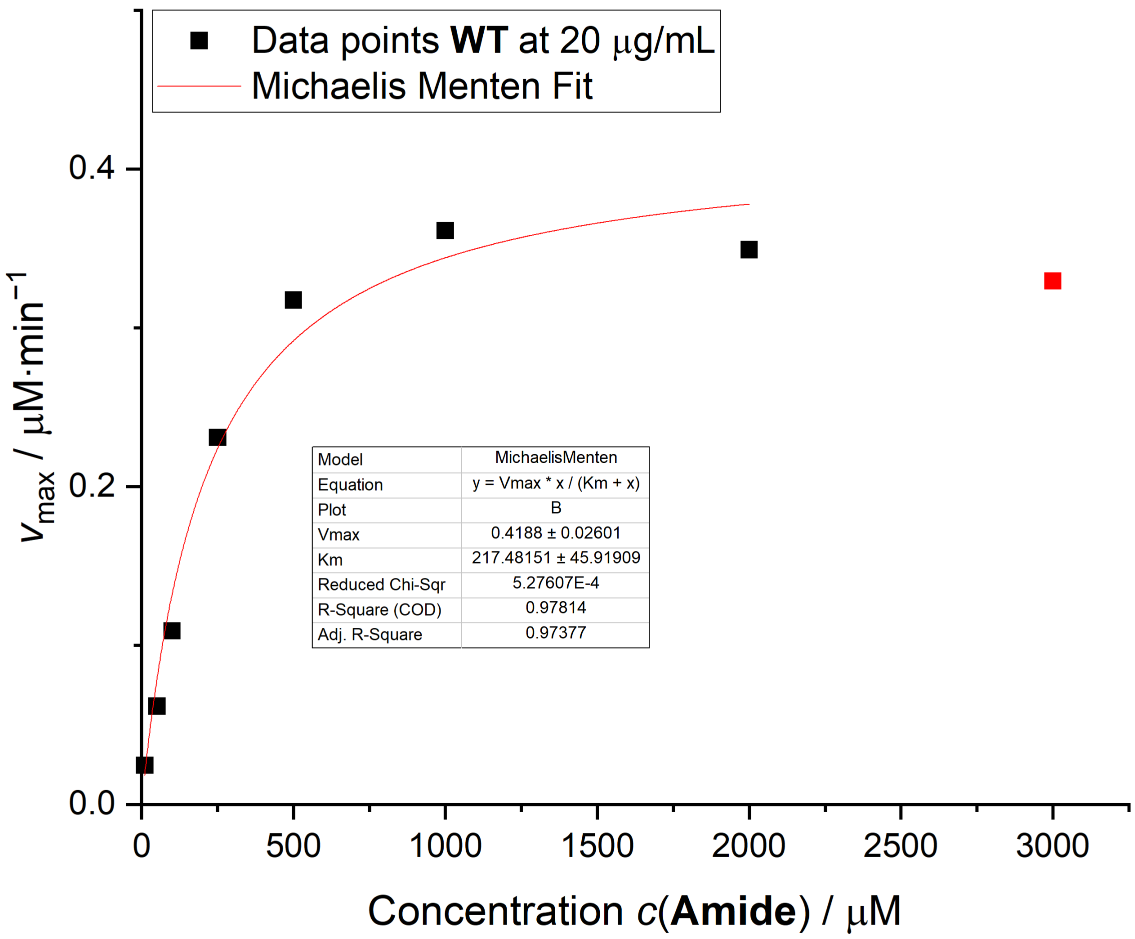

Figure S9. Michaelis–Menten plot for wild-type Bs2, run 1.

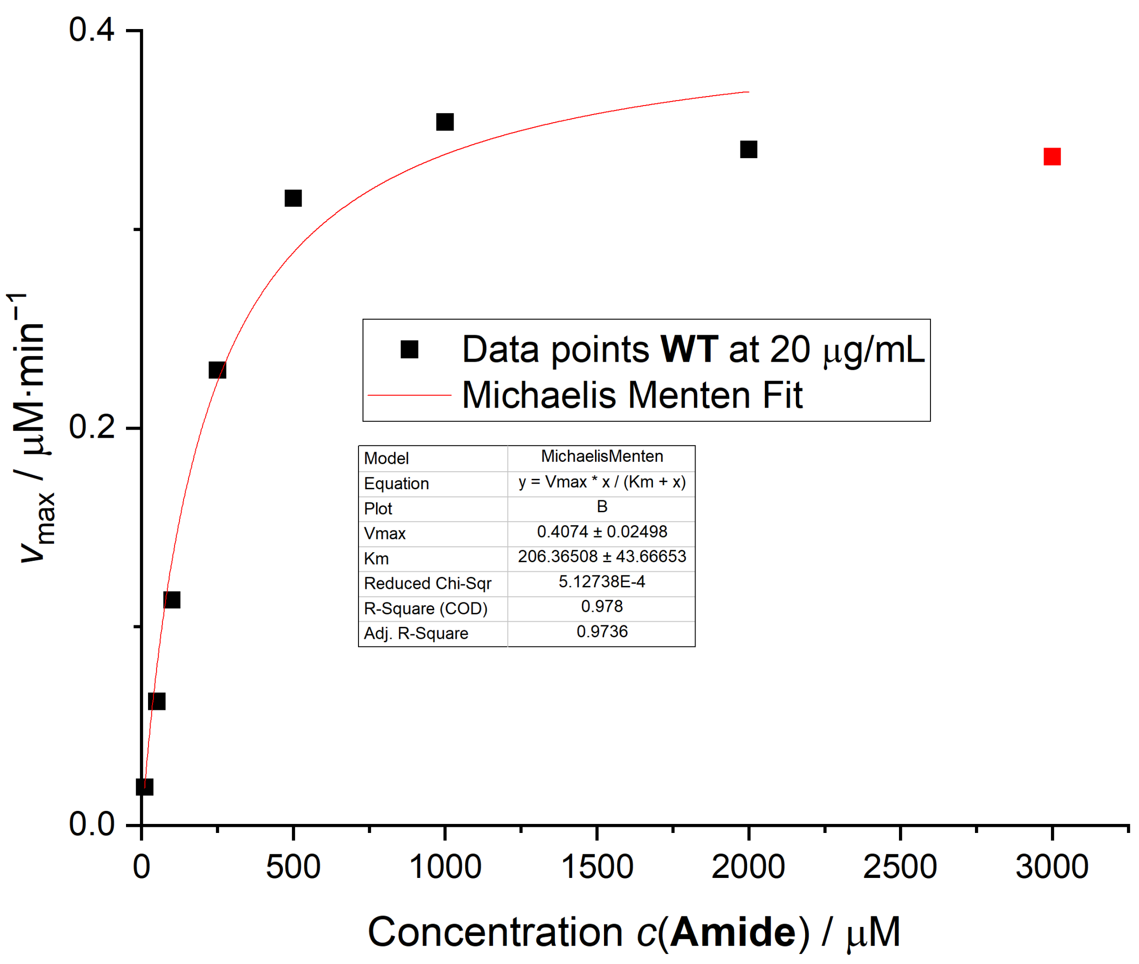

Figure S10. Michaelis–Menten plot for wild-type Bs2, run 2.

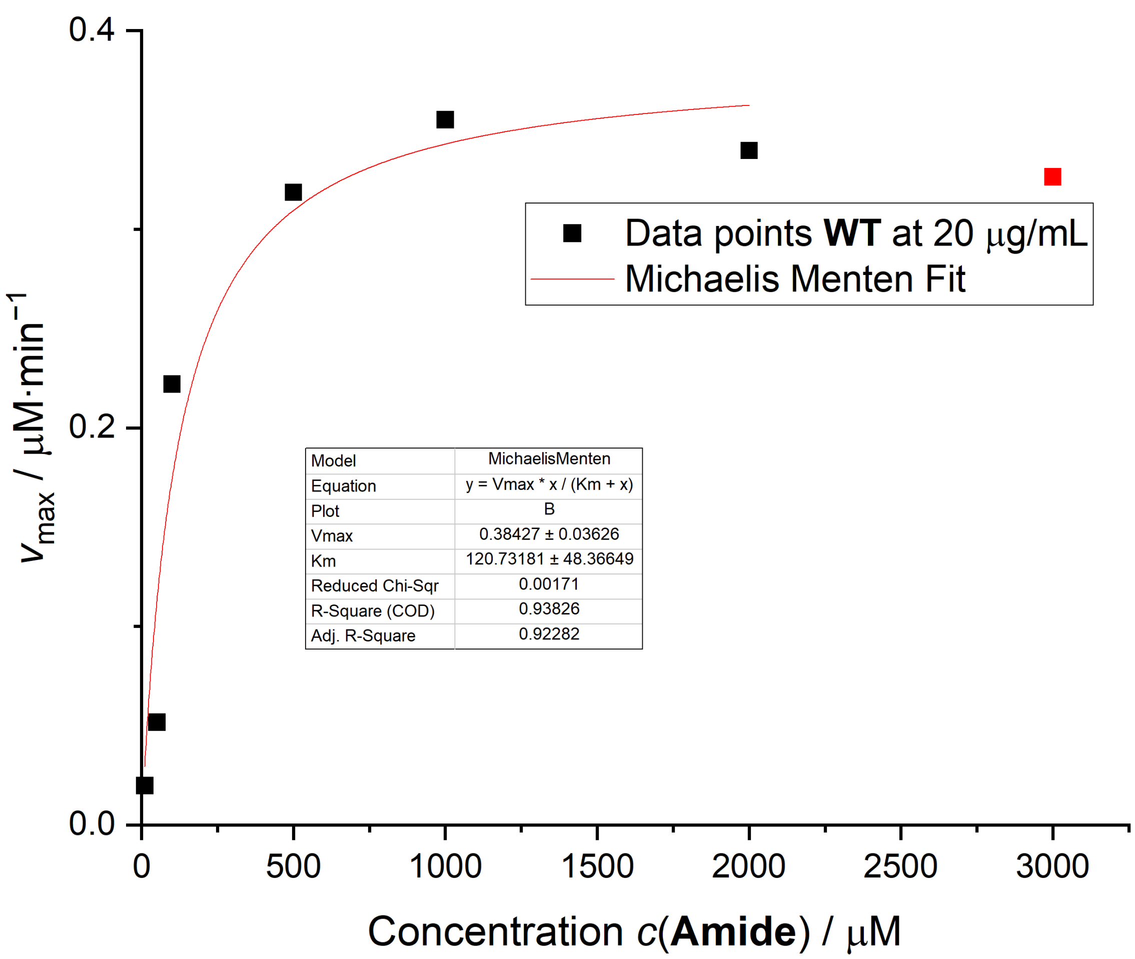

Figure S11. Michaelis–Menten plot for wild-type Bs2, run 3.

REFERENCES

(1) Needleman, S. B.; Wunsch, C. D. A General Method Applicable to the Search for Similarities in the Amino Acid Sequence of Two Proteins. *J. Mol. Biol.* **1970**, *48*, 443–453.

(2) Rice, P.; Longden, I.; Bleasby, A. EMBOSS: The European Molecular Biology Open Software Suite. *Trends Genet.* **2000**, *16*, 276–277.

(3) Henikoff, S.; Henikoff, J. G. Amino Acid Substitution Matrices from Protein Blocks. *Proc. Natl. Acad. Sci. U. S. A.* **1992**, *89*, 10915–10919.

(4) Hackenschmidt, S.; Moldenhauer, E. J.; Behrens, G. A.; Gand, M.; Pavlidis, I. V.; Bornscheuer, U. T. Enhancement of Promiscuous Amidase Activity of a Bacillus Subtilis Esterase by Formation of a π-π Network. *ChemCatChem* **2014**, *6*, 1015–1020.

(5) Consortium, T. U. UniProt: A Worldwide Hub of Protein Knowledge. *Nucleic Acids Res.* **2018**, *47*, D506–D515.

(6) Spiller, B.; Gershenson, A.; Arnold, F. H.; Stevens, R. C. A Structural View of Evolutionary Divergence. *Proc. Natl. Acad. Sci. U. S. A.* **1999**, *96*, 12305–12310.

(7) Sali, A.; Blundell, T. L. Comparative Protein Modelling by Satisfaction of Spatial Restraints. *J. Mol. Biol.* **1993**, *234*, 779–815.

(8) Olsson, M. H. M.; SØndergaard, C. R.; Rostkowski, M.; Jensen, J. H. PROPKA3: Consistent Treatment of Internal and Surface Residues in Empirical p Kapredictions. *J. Chem. Theory Comput.* **2011**, *7*, 525–537.

(9) Søndergaard, C. R.; Olsson, M. H. M.; Rostkowski, M.; Jensen, J. H. Improved Treatment of Ligands and Coupling Effects in Empirical Calculation and Rationalization of PKa Values. *J Chem Theory Comput* **2011**, *7*, 2284–2295.

(10) Jorgensen, W. L.; Chandrasekhar, J.; Madura, J. D.; Impey, R. W.; Klein, M. L. Comparison of Simple Potential Functions for Simulating Liquid Water. *J. Chem. Phys.* **1983**, *79*, 926–935.

(11) Galmés, M. A.; García-Junceda, E.; Świderek, K.; Moliner, V. Exploring the Origin of Amidase Substrate Promiscuity in CALB by a Computational Approach. *ACS Catal.* **2020**, *10*, 1938–1946.

(12) Zhang, W. E. I.; Yang, R.; Cieplak, P.; Luo, R. A. Y.; Lee, T.; Caldwell, J.; Wang, J.;

Kollman, P. A Point-Charge Force Field for Molecular Mechanics Simulations of Proteins Based on Condensed-Phase. *J. Comput. Chem.* **2003**, *24*, 1999–2012.

(13) Phillips, J. C.; Braun, R.; Wang, W. E. I.; Gumbart, J.; Tajkhorshid, E.; Villa, E.; Chipot, C.; Skeel, R. D.; Poincare, H. Scalable Molecular Dynamics with NAMD. *J. Comput. Chem.* **2005**, *26*, 1781–1802.

(14) Grest, G. S.; Kremer, K. Molecular Dynamics Simulation for Polymers in the Presence of a Heat Bath. *Phys. Rev. A* **1986**, *33*, 3628–3631.

(15) Field, M. J.; Bash, P. A.; Karplus, M. A Combined Quantum Mechanical and Molecular Mechanical Potential for Molecular Dynamics Simulations. *J. Comput. Chem.* **1990**, *11*, 700–733.

(16) Jorgensen, W. L.; Maxwell, D. S.; Tirado-Rives, J. Development and Testing of the OPLS All-Atom Force Field on Conformational Energetics and Properties of Organic Liquids. *J. Am. Chem. Soc.* **1996**, *118*, 11225–11236.

(17) Field, M. J.; Albe, M.; Bret, C.; Proust-De Martin, F.; Thomas, A. The Dynamo Library for Molecular Simulations Using Hybrid Quantum Mechanical and Molecular Mechanical Potentials. *J. Comput. Chem.* **2000**, *21*, 1088–1100.

(18) Dewar, M. J. S.; Zoebisch, E. G.; Healy, E. F.; Stewart, J. J. P. Development and Use of Quantum Mechanical Molecular Models. 76. AM1: A New General Purpose Quantum Mechanical Molecular Model. *J. Am. Chem. Soc.* **1985**, *107*, 3902–3909.

(19) Zhao, Y.; Truhlar, D. G. The M06 Suite of Density Functionals for Main Group Thermochemistry, Thermochemical Kinetics, Noncovalent Interactions, Excited States, and Transition Elements: Two New Functionals and Systematic Testing of Four M06-Class Functionals and 12 Other Function. *Theor. Chem. Acc.* **2008**, *120*, 215–241.

(20) Stewart, J. J. P. Quantum Chemistry Program Exchange 455. **1996**, *6*.

(21) Frisch, M. J.; Trucks, G. W.; Schlegel, H. B.; Scuseria, G. E.; Robb, M. A.; Cheeseman, J. R.; Scalmani, G.; Barone, V.; Mennucci, B.; Petersson, G. A.; Nakatsuji, H.; Caricato, M.; Li, X.; Hratchian, H. P.; Izmaylov, A. F.; Bloino, J.; Zheng, G.; Sonnenberg, J. L.; Hada, M.; Ehara, M.; Toyota, K.; Fukuda, R.; Hasegawa, J.; Ishida, M.; Nakajima, T.; Honda, Y.; Kitao, O.; Nakai, H.; Vreven, T.; Montgomery, J. A., Jr.; Peralta, J. E.; Ogliaro, F.; Bearpark, M.; Heyd, J. J.; Brothers,

E.; Kudin, K. N.; Staroverov, V. N.; Kobayashi, R.; Normand, J.; Raghavachari, K.; Rendell, A.; Burant, J. C.; Iyengar, S. S.; Tomasi, J.; Cossi, M.; Rega, N.; Millam, J. M.; Klene, M.; Knox, J. E.; Cross, J. B.; Bakken, V.; Adamo, C.; Jaramillo, J.; Gomperts, R.; Stratmann, R. E.; Yazyev, O.; Austin, A. J.; Cammi, R.; Pomelli, C.; Ochterski, J. W.; Martin, R. L.; Morokuma, K.; Zakrzewski, V. G.; Voth, G. A.; Salvador, P.; Dannenberg, J. J.; Dapprich, S.; Daniels, A. D.; Farkas, O.; Foresman, J. B.; Ortiz, J. V.; Cioslowski, J.; Fox, D. J. Gaussian 09, Revision E.01. Gaussian, Inc.: Wallingford, CT 2009.

(22) Byrd, R. H.; Lu, P.; Nocedal, J.; Zhu, C. A Limited Memory Algorithm for Bound Constrained Optimization. *SIAM J. Sci. Comput.* **1995**, *16*, 1190–1208.

(23) J. Turner, A.; Moliner, V.; H. Williams, I. Transition-State Structural Refinement with GRACE and CHARMM: Flexible QM/MM Modelling for Lactate Dehydrogenase. *Phys. Chem. Chem. Phys.* **1999**, *1*, 1323–1331.

(24) Martí, S.; Moliner, V.; Tuñón, I. Improving the QM/MM Description of Chemical Processes: A Dual Level Strategy to Explore the Potential Energy Surface in Very Large Systems. *J. Chem. Theory Comput.* **2005**, *1*, 1008–1016.

(25) Baker, J.; Kessi, A.; Delley, B. The Generation and Use of Delocalized Internal Coordinates in Geometry Optimization. *J. Chem. Phys.* **1996**, *105*, 192–212.

(26) Baker, J. Constrained Optimization in Delocalized Internal Coordinates. *J. Comput. Chem.* **1997**, *18*, 1079–1095.

(27) Roux, B. The Calculation of the Potential of Mean Force Using Computer-Simulation. *Comput. Phys. Commun.* **1995**, *91*, 275–282.

(28) Torrie, G. M.; Valleau, J. P. Non-Physical Sampling Distibutions in Monte-Carlo Free-Energy Estimation - Umbrella Sampling. *J. Comput. Phys.* **1977**, *23*, 187–199.

(29) Kumar, S.; Rosenberg, J. M.; Bouzida, D.; Swendsen, R. H.; Kollman, P. A. The Weighted Histogram Analysis Method for Free-energy Calculations on Biomolecules. I. The Method. *J. Comput. Chem.* **1992**, *13*, 1011–1021.

(30) Verlet, L. Computer "Experiments" on Classical Fluids. I. Thermodynamical Properties of Lennard-Jones Molecules. *Phys. Rev.* **1967**, *159*, 98–103.

(31) Ruiz-Pernía, J. J.; Silla, E.; Tuñón, I.; Martí, S.; Moliner, V. Hybrid QM/MM Potentials of Mean Force with Interpolated Corrections. *J. Phys. Chem. B* **2004**, *108*,

8427–8433.

(32) Ruiz-Pernía, J. J.; Silla, E.; Tuñón, I.; Martí, S. Hybrid Quantum Mechanics/Molecular Mechanics Simulations with Two-Dimensional Interpolated Corrections: Application to Enzymatic Processes. *J. Phys. Chem. B* **2006**, *110*, 17663–17670.

(33) UniProt: The Universal Protein Knowledgebase in 2021. *Nucleic Acids Res.* **2021**, *49*, D480–D489.

(34) Sievers, F.; Wilm, A.; Dineen, D.; Gibson, T. J.; Karplus, K.; Li, W.; Lopez, R.; McWilliam, H.; Remmert, M.; Soeding, J.; Thompson, J. D.; Higgins, D. G. Fast, Scalable Generation of High-Quality Protein Multiple Sequence Alignments Using Clustal Omega. *Mol. Syst. Biol.* **2011**, *7*.

(35) Cock, P. J. A.; Antao, T.; Chang, J. T.; Chapman, B. A.; Cox, C. J.; Dalke, A.; Friedberg, I.; Hamelryck, T.; Kauff, F.; Wilczynski, B.; de Hoon, M. J. L. Biopython: Freely Available Python Tools for Computational Molecular Biology and Bioinformatics. *BIOINFORMATICS* **2009**, *25*, 1422–1423.

(36) Sokal, R. R.; Michener, C. D.; Kansas., U. of. *A Statistical Method for Evaluating Systematic Relationships*; [University of Kansas]: [Lawrence, Kan.], 1958.

(37) Asnicar, F.; Weingart, G.; Tickle, T. L.; Huttenhower, C.; Segata, N. Compact Graphical Representation of Phylogenetic Data and Metadata with GraPhlAn. *PeerJ* **2015**, *3*.

(38) Berman, H. M.; Westbrook, J.; Feng, Z.; Gilliland, G.; Bhat, T. N.; Weissig, H.; Shindyalov, I. N.; Bourne, P. E. The Protein Data Bank. *Nucleic Acids Res.* **2000**, *28*, 235–242.

(39) Harris, C. R.; Millman, K. J.; van der Walt, S. J.; Gommers, R.; Virtanen, P.; Cournapeau, D.; Wieser, E.; Taylor, J.; Berg, S.; Smith, N. J.; Kern, R.; Picus, M.; Hoyer, S.; van Kerkwijk, M. H.; Brett, M.; Haldane, A.; del Río, J.; Wiebe, M.; Peterson, P.; Gérard-Marchant, P.; Sheppard, K.; Reddy, T.; Weckesser, W.; Abbasi, H.; Gohlke, C.; Oliphant, T.E. Array Programming with {NumPy}. *Nature* **2020**, *585*, 357–362.

(40) Virtanen, P.; Gommers, R.; Oliphant, T. E.; Haberland, M.; Reddy, T.; Cournapeau, D.; Burovski, E.; Peterson, P.; Weckesser, W.; Bright, J.; van der Walt, S. J.; Brett, M.; Wilson, J.; Millman, K. J.; Mayorov, N.; Nelson, A. R. J.; Jones, E.; Kern, R.; Larson,

E.; Carey, C.J.; Polat, I.; Feng, Y.; Moore, E.W.; VanderPlas, J.; Laxalde, D.; Perktold, J.; Cimrman, R.; Henriksen, I.; Quintero, E.A.; Harris, C.R.; Archibald, A.M.; Ribeiro, A.H.; Pedregosa, F.; van Mulbregt, P.; SciPy 1.0 Contributors. {SciPy} 1.0: Fundamental Algorithms for Scientific Computing in Python. *Nat. Methods* **2020**, *17*, 261–272.

(41) He, K.; Zhang, X.; Ren, S.; Sun, J. Deep Residual Learning for Image Recognition. In *2016 IEEE Conference on Computer Vision and Pattern Recognition (CVPR)*; 2016; pp 770–778.

(42) Kingma, D. P.; Ba, J. Adam: A Method for Stochastic Optimization. *CoRR* **2015**, *abs/1412.6*.

(43) Chollet, F.; others. Keras. GitHub 2015.

(44) Abadi, M.; Agarwal, A.; Barham, P.; Brevdo, E.; Chen, Z.; Citro, C.; Corrado, G. S.; Davis, A.; Dean, J.; Devin, M.; Ghemawat, S.; Goodfellow, I.; Harp, A.; Irving, G.; Isard, M.; Jia, Y.; Jozefowicz, R.; Kaiser, L.; Kudlur, M.; Levenberg, J.; Mane, D.; Monga, R.; Moore, S.; Murray, D.; Olah, C.; Schuster, M.; Shlens, J.; Steiner, B.; Sutskever, I.; Talwar, K.; Tucker, P.; Vanhoucke, V.; Vasudevan, V.; Viegas, F.; Vinyals, O.; Warden, P.; Wattenberg, M.; Wicke, M.; Yu, Y.; Zheng, X. TensorFlow: Large-Scale Machine Learning on Heterogeneous Distributed Systems. 2016.
